# Sequential Infiltration Synthesis of Al_2_O_3_ in Biodegradable Polybutylene Succinate: Characterization of the Infiltration Mechanism

**DOI:** 10.1021/acsapm.2c01073

**Published:** 2022-10-03

**Authors:** Alessia Motta, Gabriele Seguini, Michele Perego, Roberto Consonni, Antonella Caterina Boccia, Gina Ambrosio, Camilla Baratto, Pierfrancesco Cerruti, Marino Lavorgna, Stefano Tagliabue, Claudia Wiemer

**Affiliations:** † CNR-IMM, Unit of Agrate Brianza, Via C. Olivetti 2, I-20864 Agrate Brianza, Italy; ‡ Department of Energy, Politecnico di Milano, Via Ponzio 34/3, 20133 Milano, Italy; § CNR-SCITEC, Via A. Corti 12, I-20133 Milano, Italy; ∥ CNR-INO, PRISM Lab, Via Branze 45, 25123 Brescia, Italy; ⊥ CNR-IPCB, Via G. Previati 1/E, 23900 Lecco, Italy; # Corapack srl, Via del Fontaline 7, 22040 Brenna, Italy

**Keywords:** biopolymers, hybrid materials, Al_2_O_3_, TMA, SIS, packaging, NMR spectroscopy

## Abstract

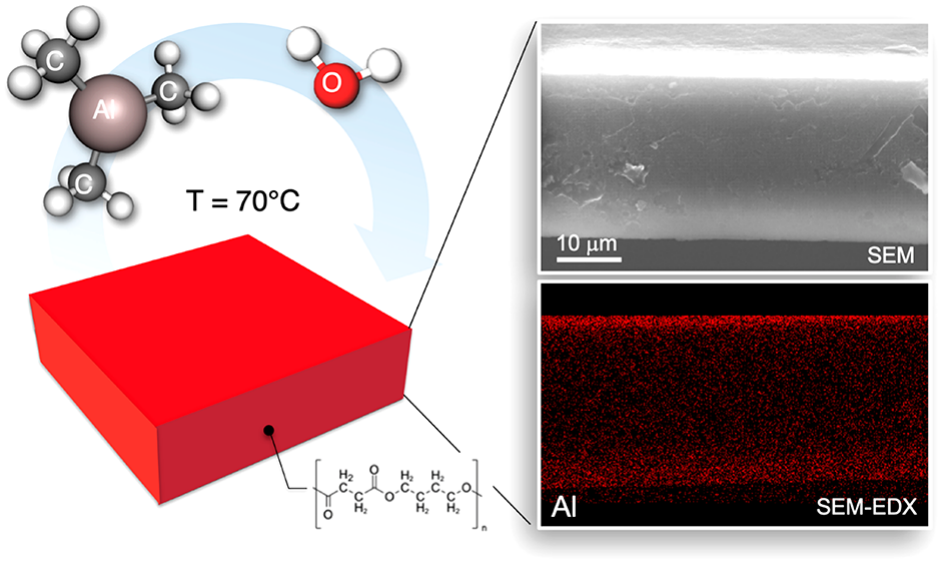

The introduction of inorganic materials into biopolymers has been envisioned as a viable option to modify the optical and structural properties of these polymers and promote their exploitation in different application fields. In this work, the growth of Al_2_O_3_ in freestanding ∼30-μm-thick poly(butylene succinate) (PBS) films by sequential infiltration (SIS) at 70 °C via trimethylaluminum (TMA) and H_2_O precursors was investigated for the first time. The incorporation of Al_2_O_3_ into the PBS matrix was clearly demonstrated by XPS analysis and SEM-EDX cross-sectional images showing a homogeneous Al_2_O_3_ distribution inside the PBS films. Raman measurements on infiltrated freestanding PBS show a reduction of the signal related to the ester carbonyl group as compared to pristine freestanding PBS films. Accordingly, FTIR and NMR characterization highlighted that the ester group is involved in polymer–precursor interaction, leading to the formation of an aliphatic group and the concomitant rupture of the main polymeric chain. Al_2_O_3_ mass uptake as a function of the number of SIS cycles was studied by infiltration in thin PBS films spin-coated on Si substrates ranging from 30 to 70 nm. Mass uptake in the PBS films was found to be much higher than in standard poly(methyl methacrylate) (PMMA) films, under the same process conditions. Considering that the density of reactive sites in the two polymers is roughly the same, the observed difference in Al_2_O_3_ mass uptake is explained based on the different free volume of these polymers and the specific reaction mechanism proposed for PBS. These results assessed the possibility to use SIS as a tool for the growth of metal oxides into biopolymers, paving the way to the synthesis of organic–inorganic hybrid materials with tailored characteristics.

## Introduction

The use of plastics is widespread in everyday life due to their versatility, strength to weight ratio, and cost. However, plastics are responsible for soil, atmosphere, and marine system pollution that has been even worsened by the recent coronavirus pandemic (COVID-19).^1^ In particular, more than 90% of plastic materials are petroleum-based and nonbiodegradable,^2^ and this leads to cumulative environmental damage caused by nondegradable plastics in the form of microplastic pollution,^3^ loss of soil fertility,^4^ and impact on wildlife.^5^ Without appropriate plastic waste management, the release of toxic chemicals as well as microplastic production are expected to further increase.^6^ In this respect, the development of biodegradable polymeric materials represents a simple and viable solution to reduce the environmental footprint due to the increasing utilization of single-use plastics.^7^

**Figure 1:**
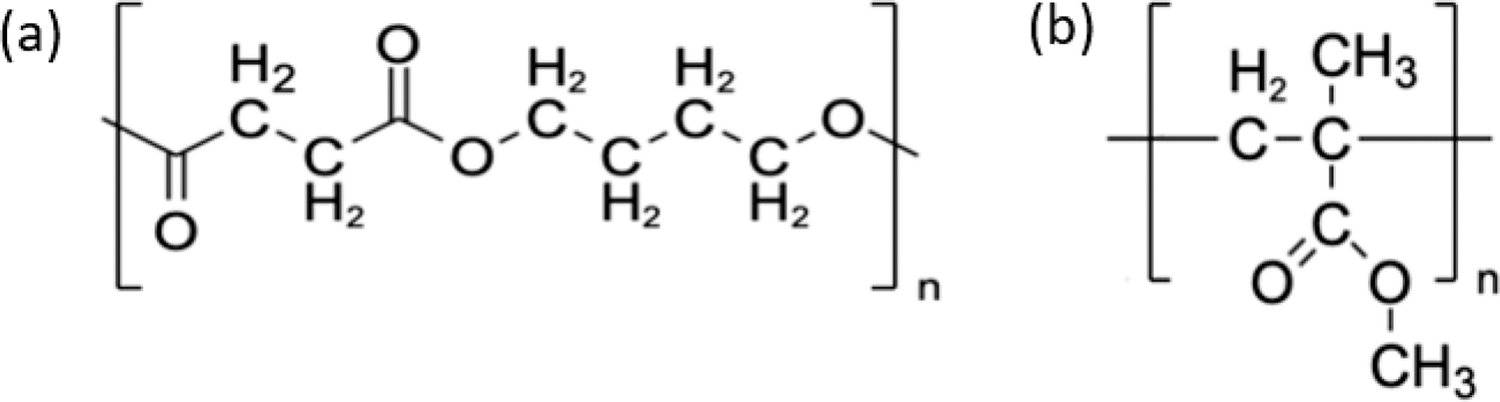
Chemical structures of PBS (a) and PMMA (b) polymers, highlighting the different position of the carbonyl groups in the two polymers.

Among commercially available biodegradable polymers, poly(butylene succinate) (PBS) is considered an emerging material. PBS is an aliphatic thermoplastic polyester (Figure 1a) with good mechanical properties that are comparable to polyethylene (PE) and polypropylene (PP). PBS shows high thermal and chemical resistance and high heat deflection temperature.^8^ Moreover, PBS exhibits excellent processability, since it can be processed with many different techniques such as film blowing, fiber spinning, injection molding, thermoforming, and blow molding.^9^ For all these reasons, it is considered a promising polymer for various potential applications including packaging, mulching films, shopping, trash bags, and disposable food containers.^10^ However, other properties of PBS, such as softness, gas barrier properties, and melt viscosity may limit its practical use.^11^ To overcome these problems, the introduction of an inorganic filler could help to expand the range of applications of this polymer. For example, the introduction of ZnO inorganic filler^12^ by dry-mixing leads to an increase of the tear strength and retards the microbiological corrosion.

Organic–inorganic hybrid materials have attracted a lot of interest in the scientific community, and several approaches have been developed to synthesize them and tailor their properties. Usually these hybrid materials are synthesized by liquid solution methods.^13−15^ In the past decade, vapor phase processes have emerged as an alternative technique for the incorporation of an inorganic component into an organic matrix to develop hybrid materials.^16^ For example, a thin Al_2_O_3_ top coating layer grown by atomic layer deposition (ALD) on a polymeric substrate could work as a high-quality pore-free barrier film.^17^ Among vapor phase processes, sequential infiltration synthesis (SIS) has emerged as a promising process to synthesize hybrid materials starting from a polymeric template.^18,19^ SIS is a vapor phase deposition technique that implies the sequential exposure of the organic materials to vapors of an organometallic precursor and a coprecursor, intercalating the two exposure steps by appropriate purging cycles of inert gas to remove unreacted molecules or reaction byproducts. During exposure, the precursor diffuses into the organic matrix and remains entrapped inside the material due to interaction between precursors and reactive sites inside the organic matrix. These nucleation sites act as seeds for the subsequent growth of the inorganic materials within the polymer. Actually, SIS is a very complex process that involves the chemical and physical phenomena of the precursors inside the polymer matrix. As a consequence, process parameters must be optimized taking into account the chemical and physical properties of the polymer that has been selected as the organic matrix. So far, SIS has been tested to generate hybrid materials for numerous different applications including advanced lithography,^20^ oil sorbent,^21^ ultrafiltration,^22^ organic solvent,^23^ gas separation,^24^ membranes, optical coatings,^25^ and optoelectronics.^26^

In this work, infiltration of Al_2_O_3_ into freestanding ∼30-μm-thick PBS films was established by means of a SIS process at 70 °C using trimethylaluminum (TMA) and H_2_O precursors. Al_2_O_3_ mass uptake was studied at an increasing number of SIS cycles as a function of the initial polymer thicknesses of thin PBS films spin-coated on Si substrates. A comparison with poly(methyl methacrylate) (PMMA) thin films was applied for a better understanding of the process mechanism. Accordingly, pristine and infiltrated freestanding PBS films were characterized by Raman, Fourier-Transform Infrared (FTIR), and Nuclear Magnetic Resonance (NMR) spectroscopies, and a reasonable reaction mechanism was hypothesized. UV shielding properties of the infiltrated PBS films as well as their thermal stability were investigated. These experimental results highlight the possible limitations of the proposed approach and suggest a viable approach to enable the design of hybrid materials by SIS on biopolymers.

## Experimental Methods

### Materials

PBS in the form of freestanding film (30 μm thick) was provided by Corapack. Additionally, PBS and PMMA films with thickness below 100 nm were formed on Si wafers by spin coating a solution of the dissolved polymer at 3000 rpm and room temperature. More specifically, PBS was dissolved in chloroform, and PMMA (*M*_n_ = 14 kg mol^–1^, polydispersity index (PDI) = 1.2) was dissolved in toluene, properly adjusting the concentration to obtain different polymeric film thicknesses. Before PBS casting, the Si substrates were cleaned with 2-propanol in an ultrasonic bath and dried under N_2_. Before PMMA casting, the Si substrates were cleaned in Piranha solution (H_2_SO_4_:H_2_O_2_ = 3:1 v/v) at 80 °C for 40 min, rinsed with 2-propanol in an ultrasonic bath, and dried with a N_2_ stream for PMMA films. The PBS films were infiltrated without any additional thermal treatment. Conversely, before infiltration, the PMMA films were annealed at 250 °C for 900 s in N_2_ atmosphere by means of a Rapid Thermal Process (RTP) tool in order to remove residual toluene from the spun film.

### Sequential Infiltration Synthesis

Samples were loaded in a commercial cross-flow ALD reactor (Savannah 200, Ultratech Cambridge NanoTech.) and thermalized at 70 °C for 30 min under 100 sccm N_2_ flow at 0.6 Torr before starting the infiltration. All the infiltrations were performed at 70 °C, because higher temperatures were observed to induce PBS degradation and sticking on the supporting wafer. Furthermore, Raman spectroscopy analysis confirmed the absence of thermal degradation effects in the vibrations of PBS treated at 70 °C (Figure S1). During SIS of Al_2_O_3_, polymer samples were alternatively exposed to TMA (Aldrich, 97%) and deionized H_2_O. Exposures of the polymer samples to precursor vapors were intercalated by a purge phase in N_2_ flow at 100 sccm. In more detail, the SIS cycle was composed of TMA exposure/N_2_ purge/H_2_O exposure/N_2_ purge. The number of SIS cycles was varied between 5 and 25, and TMA pulse time was varied between 0.025 and 0.040 s. Before the exposure steps, the valve to the pump was closed and the nitrogen flux set to 0 in order to create static vacuum inside the deposition chamber before the introduction of the precursors. TMA exposure time (60 s), H_2_O exposure time (60 s), TMA purge time (60 s), and H_2_O purge time (300 s) were kept constant. The evolution of the total pressure in the growth chamber during the SIS process is shown in Figure S2 at different TMA pulse times in order to facilitate the repetition of the infiltration experiments. It is important to note that the pressure is significantly affected by the amount of material inside the growth chamber. Upon the SIS process, the thin film samples were exposed to O_2_ plasma to remove the polymer matrix, obtaining alumina thin films deposited on the silicon substrate.

Additionally, Al_2_O_3_ growth on PBS films was performed by 115 ALD cycles at 70 °C. The precursors were introduced in the reactor in alternate pulses, separated by an inert N_2_ gas pulse. More specifically the ALD cycle is composed of four process steps: (i) TMA pulse (0.015 s), (ii) N_2_ purge pulse (20 s), (iii) water pulse (0.015 s), and (iv) N_2_ purge pulse (30 s).

### Characterization Techniques

Pristine and infiltrated polymer samples were characterized by spectroscopic ellipsometry and XPS analysis to monitor Al_2_O_3_ incorporation into PBS. Raman, FTIR, and NMR spectroscopies were performed to obtain information about the reaction mechanism. The effect of the Al_2_O_3_ infiltration on the thermal properties of the pristine and infiltrate PBS films was evaluated by differential scanning calorimetry (DSC) and thermogravimetric (TG) analysis. UV–vis spectrophotometry was performed to investigate optical properties of pristine and infiltrated films. Detailed information about these characterization techniques is reported in the Supporting Information.

## Results

### Al_2_O_3_ Incorporation into a PBS Matrix

The incorporation of Al_2_O_3_ into the freestanding PBS matrix upon 5 SIS cycles at 70 °C was studied as a function of TMA pulse time. TMA pulse times (*t*_TMA_) ranging from 0.025 to 0.040 s were considered to fill the reaction chamber with precursor vapors and to provide a reservoir of molecules for diffusion into the polymer during exposure. Collected data clearly highlight the modification of the PBS matrix upon SIS. To demonstrate effective incorporation of Al_2_O_3_ into the PBS matrix and provide information about the Al_2_O_3_ depth distribution, XPS and SEM-EDX analyses were performed on the PBS samples after 5 SIS cycles with *t*_TMA_ values of 0.025 and 0.040 s, respectively. Figure S3 shows the XPS spectra of the pristine and infiltrated freestanding PBS films. The spectrum of the pristine PBS sample exhibits only two main peaks at ∼385 eV and ∼530 eV corresponding to C 1s and O 1s core levels, respectively. Conversely, the spectra of the infiltrated samples show the presence of Al core level signals at ∼120 eV and ∼75 eV corresponding to Al 2s and Al 2p core levels, respectively. These signals provide a clear indication of Al_2_O_3_ incorporation after 5 SIS cycles for both samples. The intensity of these signals is roughly the same in the two samples, irrespectively of TMA pulse time. This fact indicates that the amount of Al_2_O_3_ incorporated in the superficial PBS layer is similar. The limited escape depth of the photoelectrons prevents the acquisition of information about the depth distribution of Al_2_O_3_ into the PBS matrix.

**Figure 2:**
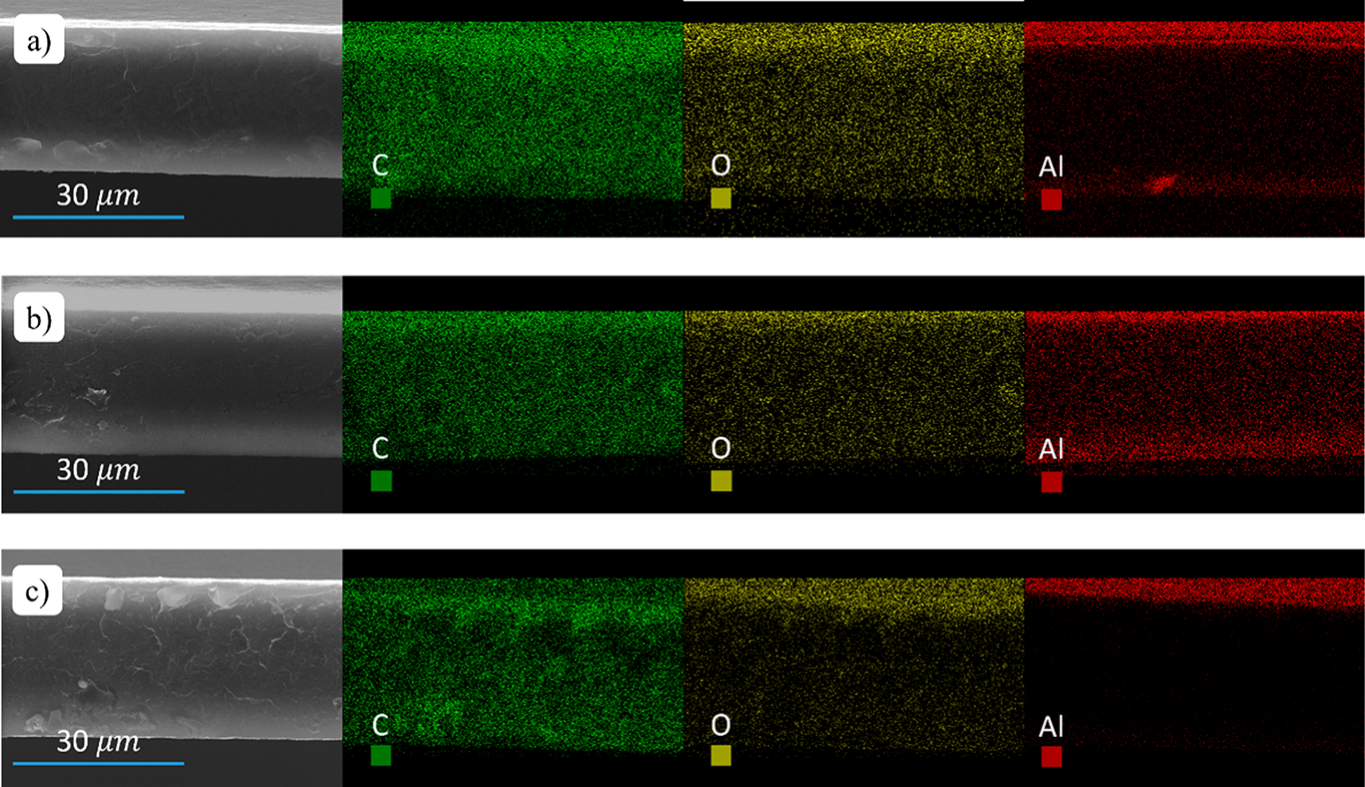
SEM and EDX elemental mapping of freestanding PBS samples upon infiltration of Al_2_O_3_ by means of 5 (a) and 25 (b) SIS cycles using the same TMA pulse time of 0.040 s SEM and EDX elemental mapping of freestanding PBS samples upon ALD growth of Al_2_O_3_ at 70 °C (c).

Figure 2a,b shows representative SEM-EDX cross-sectional images of PBS films infiltrated at 70 °C with 5 and 25 SIS cycles, respectively. TMA pulse time is 0.040 s for both samples. The elemental mapping indicates the presence of C, O, and Al. Uniform distributions of C and O are observed throughout the entire PBS film thickness for all the samples, consistent with the chemical structure of the PBS macromolecules (Figure 1a). Al elemental mapping indicates that this element is distributed throughout the entire polymeric film thickness, with some evidence of Al accumulation at the surface of the PBS sample infiltrated with 5 SIS cycles. It is worth remembering that, to effectively infiltrate the polymeric template, TMA and H_2_O precursors are expected to diffuse inside the PBS films during the hold step of the SIS process.^27^ The penetration depth of metal and oxygen precursors during SIS strongly depends on their diffusivity into the polymer matrix. Even if these data do not provide a direct measurement of TMA diffusivity, they clearly indicate that TMA and H_2_O diffusivities in PBS are extremely high at the selected processing temperature, since during the hold time they can move throughout the entire film and react with the PBS molecules. Figure 2c shows SEM-EDX cross-sectional images of an Al_2_O_3_ film that was grown on a PBS film by ALD at 70 °C. The number of ALD cycles and the processing parameter were adjusted to obtain an Al_2_O_3_ film with nominal thickness of 10 nm. In this case, O and Al distributions indicate the presence of a very high concentration of Al_2_O_3_ in the region close to the surface of the PBS sample. The surface and subsurface growth of Al_2_O_3_ into the PBS matrix is due to the limited time for the precursors to diffuse into the polymer bulk during the ALD process.^28^ Interestingly, the subsurface growth of Al_2_O_3_ during ALD further corroborates the view of extremely high TMA and H_2_O diffusivities in PBS.

Collected data indicate that TMA diffusivity does not represent a limitation for Al_2_O_3_ incorporation in PBS films during the SIS process. Accordingly, the amount of Al_2_O_3_ incorporated in the PBS matrix is expected to be proportional to the PBS volume and to the number of reactive sites into the polymer matrix. To obtain quantitative information about mass uptake, thin PBS films were spin-cast on a silicon substrate and subsequently infiltrated at 70 °C with TMA and H_2_O. Three different sets of PBS films having different thicknesses were prepared. Spinning parameters were adjusted to obtain 30-, 50-, and 70-nm-thick PBS films. The actual thickness of each PBS film was measured by spectroscopic ellipsometry. The results of the fitting of the ellipsometric data for each sample are reported in the Supporting Information (Figure S4). The average thickness of the PBS films was found to be 29.1 ± 0.3, 48.7 ± 0.4, and 68.6 ± 1.0 nm for the different sets of samples, respectively, with a very limited variation from sample to sample. After infiltration, the polymer matrix was removed by O_2_ plasma treatment, leaving an Al_2_O_3_ film on the surface of the Si substrate. Finally, the thickness of the Al_2_O_3_ film was measured by ellipsometry to obtain information about Al_2_O_3_ mass uptake.

**Figure 3:**
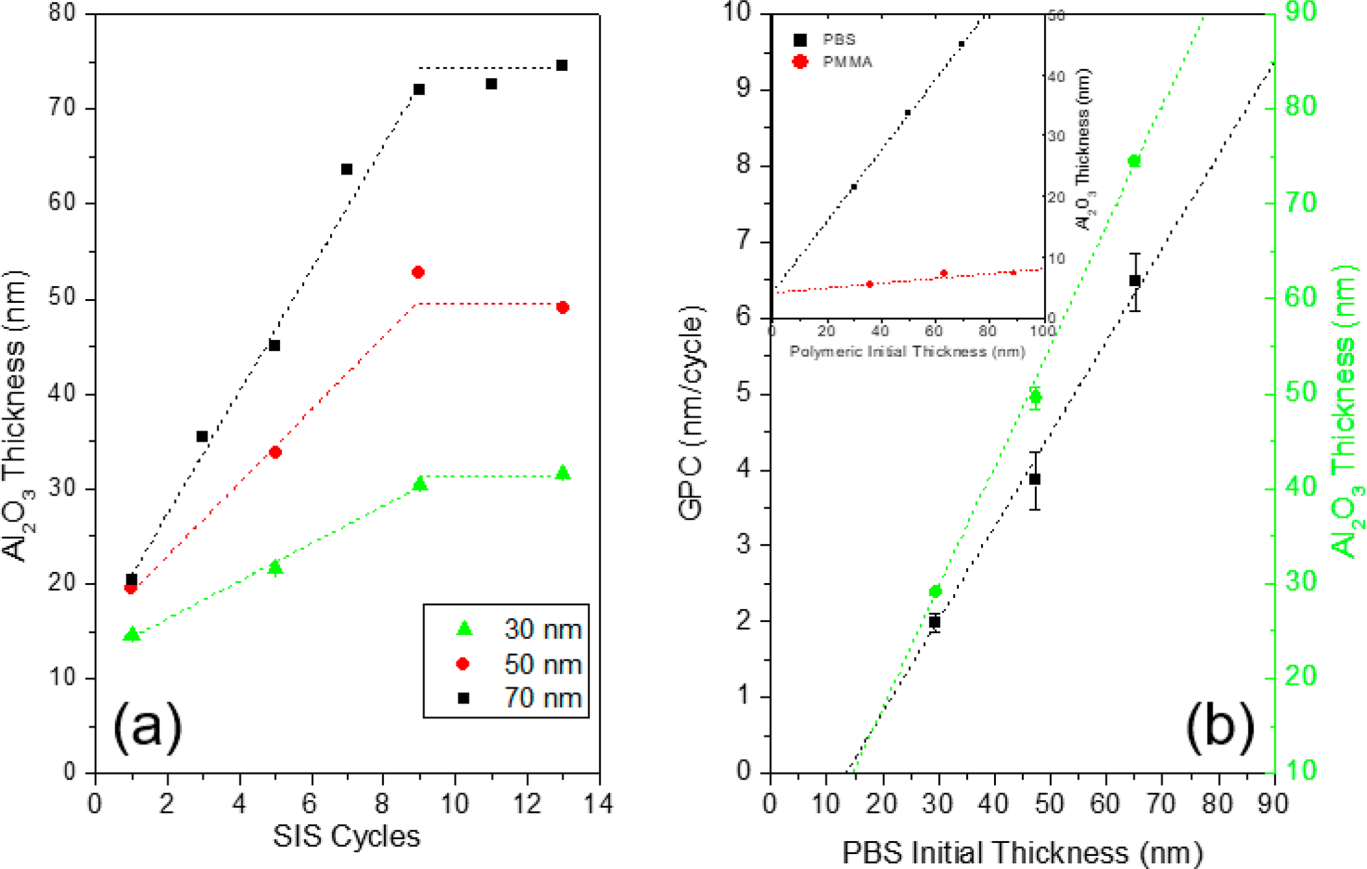
(a) Thickness of Al_2_O_3_ film obtained by infiltration in 30-, 50-, and 70-nm-thick PBS thin films and subsequent removal of the organic phase by O_2_ plasma treatment. Data are reported as a function of the number of SIS cycles. (b) Growth per cycle (GPC) and alumina film thickness as a function of the PBS film thickness. In the inset, the thicknesses of the Al_2_O_3_ layers resulting from PBS and PMMA thin films infiltrated by 5 SIS cycles under the same processing conditions are reported as a function of the initial thickness of the polymer films. The data were collected upon O_2_ plasma treatment to ash the organic part.

Figure 3a shows the evolution of Al_2_O_3_ film thickness as a function of the number of the SIS cycles for the three sets of PBS thin films having different initial thicknesses. All the samples, irrespective of the initial PBS film thickness, exhibit the same evolution of the Al_2_O_3_ film thickness which rapidly increases up to 9 SIS cycles and subsequently levels off, entering a sort of saturation regime. In particular, the initial trend is marked by a linear increase of Al_2_O_3_ film thickness. By fitting the experimental data, it is possible to obtain information about the growth per cycles (GPC); i.e., the thickness increases at each SIS cycle. Accordingly, the GPC is determined to be 2.0 ± 0.1, 3.9 ± 0.4, and 6.5 ± 0.4 nm/cycle in the 30-, 50-, and 70-nm-thick PBS films, respectively. In Figure 3b, the GPC values during the initial stages of the SIS process and the Al_2_O_3_ film thickness in the saturation regime are reported as a function of the initial thickness of the PBS films. Interestingly, the GPC and the saturation thickness values increase linearly with the PBS film thickness, further supporting the idea that the incorporation of Al_2_O_3_ is essentially limited by the number of reactive sites that are available in the PBS matrix.

From a more general point of view, it is worth noting that the amount of Al_2_O_3_ trapped in the PBS film is very high compared to other polymers using similar SIS processes. The graph in the inset of Figure 3b compares the thicknesses of the Al_2_O_3_ films obtained in PBS and PMMA thin films upon 5 SIS cycles at 70 °C with TMA and H_2_O precursors and subsequent removal of the polymer matrix by O_2_ plasma treatment. Data are reported as a function of the initial thickness of the polymer films. Both polymers exhibit a linear increase of the Al_2_O_3_ film thickness. However, the amount of Al_2_O_3_ incorporated in the PBS films is much higher than in the PMMA ones. This difference could be explained by assuming that TMA diffusivity at 70 °C in PBS is much higher than in PMMA. Collected data demonstrate that TMA can diffuse very fast in the PBS matrix. Conversely, literature data at 90 °C indicate that TMA diffusion in PMMA is quite slow.^29^ Reactivity and density of the chemical groups of the polymer chain that are involved in the SIS process could play a role in explaining these results. More information about reactive groups in the PBS matrix is necessary to understand polymer–precursor interactions and investigate the reaction mechanism during the SIS process.

### Investigation of Reaction Chemistry

Raman, FTIR, and NMR techniques were employed on pristine and infiltrated PBS samples to identify the reactive groups that lead to alumina seed formation, and to provide a tentative model for the reaction mechanism.

**Figure 4:**
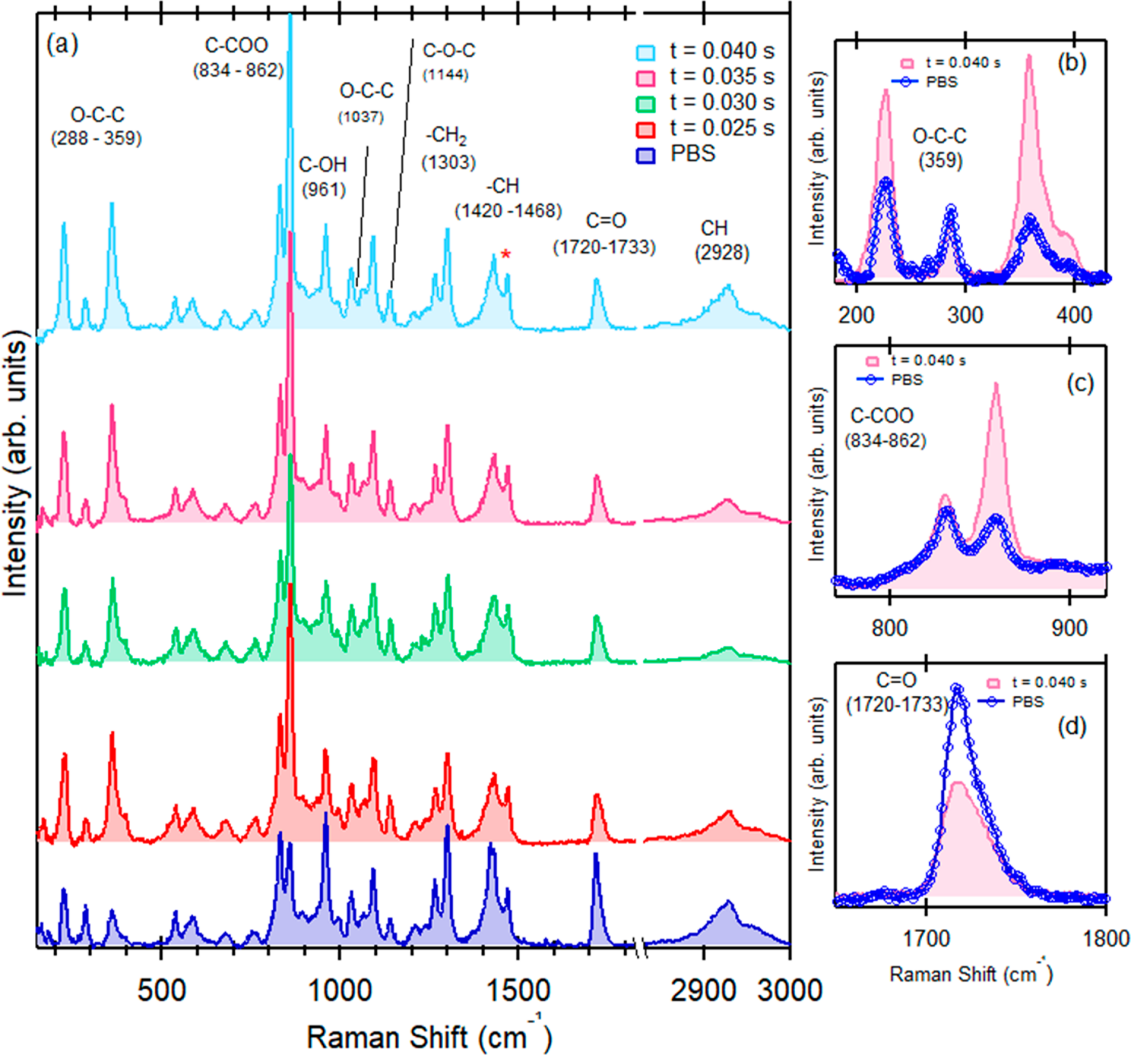
(a) Raman spectra of pristine and infiltrated PBS at different TMA pulse times (*t*_TMA_) ranging from 0.025 to 0.040 s. The number of SIS cycles is 5 for all the samples. The peak used for normalization of the spectra is indicated by *. Spectral regions corresponding to O–C–C (b), C–COO (c), and C=O (d)) for pristine PBS and for the same sample upon infiltration with TMA and H_2_O at 70 °C using *t*_TMA_ = 0.040 s. Data are obtained from the Raman spectra in panel (a) to highlight the main difference after the infiltration.

Figure 4 shows Raman spectra of the pristine and infiltrated freestanding PBS samples. The number of SIS cycles is fixed to 5 for all the samples. All the spectra were normalized to the peak of asymmetric −CH bending at 1468 cm^–1^ which is indicated by a * in Figure 4, because the C–H group is not directly involved in the infiltration process. The band assignments are in perfect agreement with the literature data.^30,31^ The comparison between the spectrum of the pristine PBS matrix and those of the infiltrated PBS samples highlights that infiltration of PBS with TMA and H_2_O precursors did not cause the formation of new peaks that can be directly associated with alumina phases, irrespective of the TMA pulse time. According to the literature, Raman signals are expected to appear at 1180 and 1247 cm^–1^ for θ-Al_2_O_3_ and at 1375 and 1405 cm^–1^ α-Al_2_O_3_.^32,33^ Actually, no peaks are present in the spectra at these specific wavelengths. Conversely, the infiltrated samples exhibit significant differences in the intensities of some carbon related peaks with respect to the spectrum of the pristine PBS matrix. In addition, by analyzing the modifications that occurred in Raman spectra of infiltrated PBS samples, it can be affirmed that different TMA pulse times do not alter the response of the polymeric film. Figure S5 reports the intensities of the more significant Raman peaks as a function of the TMA pulse times. Only the C–COO signal exhibits a significant variation when increasing the TMA pulse time.

Spectral regions exhibiting the most significant variations are shown in detail in Figure 4b,c,d, comparing the spectra of pristine PBS sample and infiltrated PBS samples. Since all the infiltrated PBS samples exhibit similar spectra irrespective of the TMA pulse times, in these figures only the spectrum of the infiltrated sample with *t*_TMA_ = 0.040 s is reported. In particular, the peak intensity of the bending vibrations along the −C–C–O– backbone vibration, at 288 cm^–1^, is increased (Figure 4b). Moreover, the intensity ratio of the peaks at 862 and 834 cm^–1^ ascribed to the ester stretching mode C–COO^34^ is doubled in the infiltrated PBS with respect to the pristine PBS as shown in the inset of Figure 4c. Finally, in the region of the ester carbonyl group (C=O) stretching vibrations (Figure 4d), peaks at 1720 and 1733 cm^–1^ are observed as clear modification in the peak intensity ratio after infiltration. No modification was observed for the peak at 961 cm^–1^ corresponding to the C–OH bending in the carboxylic acid groups of the PBS and for the peak around 1037 cm^–1^ ascribed to O–C–C stretching vibrations and for the peaks in the range of 1144–1260 cm^–1^ resulting from the stretching of the C–O–C group in the ester linkages of PBS. The peaks at 1303 and 2928 cm^–1^ are assigned to the symmetric and asymmetric deformational vibrations of the methylene groups (CH_2_) in the PBS main chains, respectively. Interestingly, these peaks did not show any significant modification. Thus, Raman spectroscopy showed marked changes in vibration modes linked to carbon–oxygen bond, indicating that this specific group could be involved in the reaction of the PBS matrix with the TMA molecules. It is worth noting that this modification is already observed at the lower pulse time, corresponding to *t*_TMA_ = 0.025 s.

**Figure 5:**
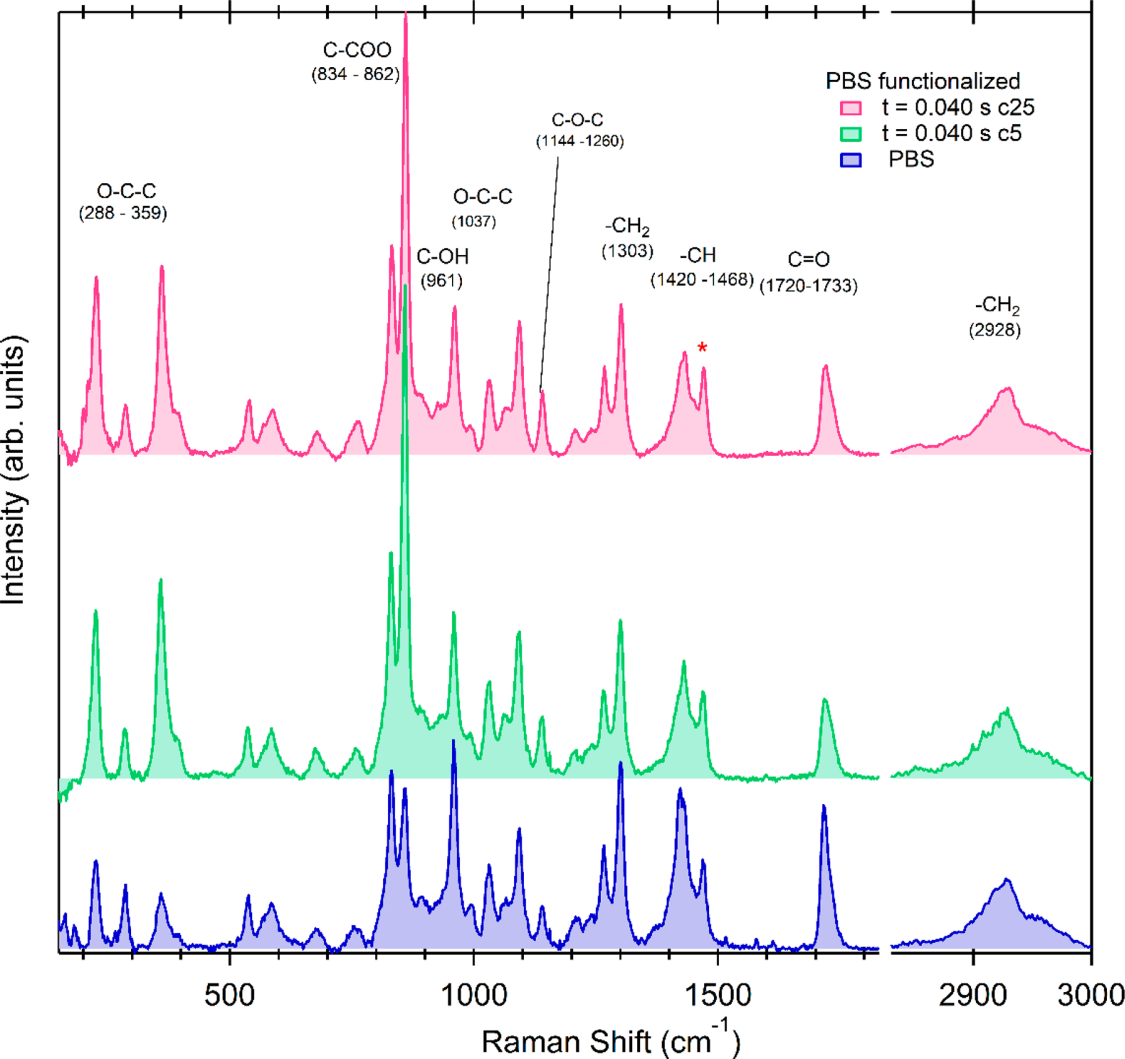
Raman spectra of pristine and infiltrated PBS by using the different number of SIS cycles. The spectrum of the sample at 5 cycles is the same as reported in Figure 1.

In order to better elucidate these effects a new set of samples was prepared, keeping constant the TMA pulse time (*t*_TMA_ = 0.040 s) and varying the number of SIS cycles. Figure 5 shows the Raman spectra for the PBS samples infiltrated with 5 and 25 SIS cycles, respectively. These spectra are compared with the spectrum of the pristine PBS film. Interestingly, the spectra modifications for PBS samples after the SIS process with respect to pristine PBS are similar to those observed in the spectra shown in Figure 4, irrespective of the number of SIS cycles.

**Figure 6:**
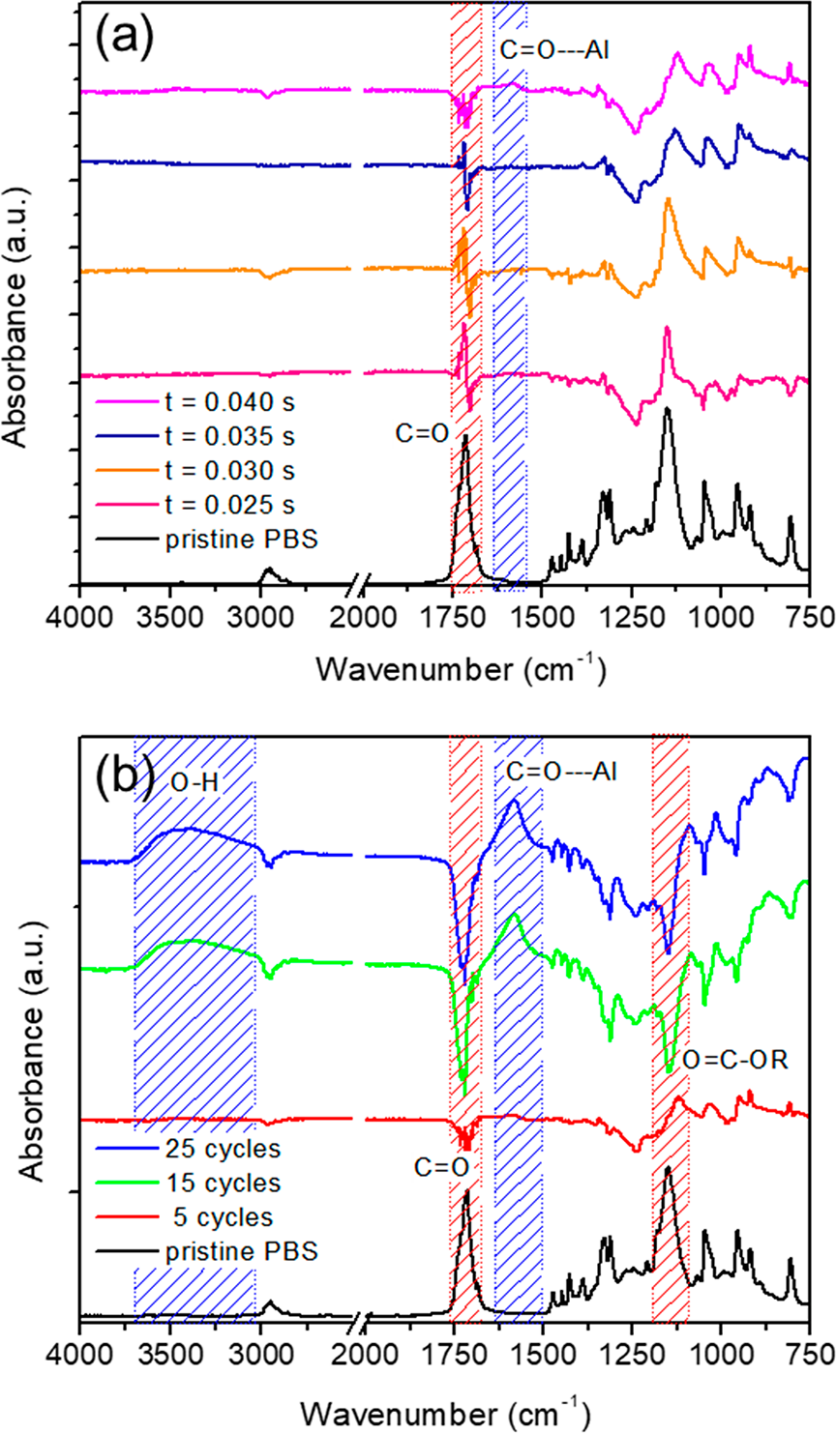
Differential FTIR-ATR spectra of infiltrated PBS film as a function of (a) TMA pulse time and (b) number of cycles. Spectra are referenced to pristine PBS film.

Figure 6 shows the FTIR–ATR spectrum of pristine PBS and the differential spectra after Al_2_O_3_ SIS process. The FTIR spectrum of pristine PBS film shows characteristic peaks at 2950 and 1328 cm^–1^, corresponding to the symmetric and asymmetric deformational vibrations of the −CH_2_– group in the PBS main chains, respectively.^35−37^ The band at 1715 cm^–1^ is attributed to the C=O stretching vibrations of the ester group in PBS. Finally, the broad peaks at 1150 cm^–1^ correspond to the −C–O–C– stretching in the ester linkages of PBS. Figure 6a illustrates the FTIR-ATR differential spectra of infiltrated PBS films obtained upon 5 SIS cycles with TMA pulse times increasing from 0.025 to 0.040 s. Slight differences between the FTIR-ATR spectra are detected at different pulse times. In particular, a blue shift of the ester C=O peak and an increase in the −C–O–C– absorbance of PBS are noted for short pulse times, likely due to the thermal treatment which perturbed the crystalline phase of PBS.^38^ A further increase in pulse duration induces a decrease of C=O and −C–O–C– absorbance, as well as the appearance of a weak 1580 cm^–1^ absorption. These features are perfectly consistent with the formation of Al–O–C– bonding units which cause the consumption of the PBS carbonyls.^39−41^Figure 6b shows FTIR-ATR spectra at the increasing number of SIS cycles at the same TMA pulse time of 0.040 s. After 15 and 25 infiltration cycles, C=O and −C–O–C– absorbance decreases, accompanied by the dramatic buildup of the Al–O–C peaks at 1580 cm^–1^. Additionally, the O–H band at 3400 cm^–1^ increase by 2 and 3 times, respectively (Figure S6). The latter absorption stems from the hydration of the surfaces of alumina formed within the polymer bulk.^42^ Finally, a decrease in the absorbance of the −CH_2_– groups at 2950 and 1328 cm^–1^ is also noticed, due to the change in the vibrational mode of these groups. According to the FTIR-ATR analysis, the different TMA pulse times do not change the response of the polymeric film, in good agreement with previously discussed Raman spectra. Additionally, FTIR-ATR analysis further supports the hypothesis that the esters are the reactive groups in the PBS matrix.

**Figure 7:**
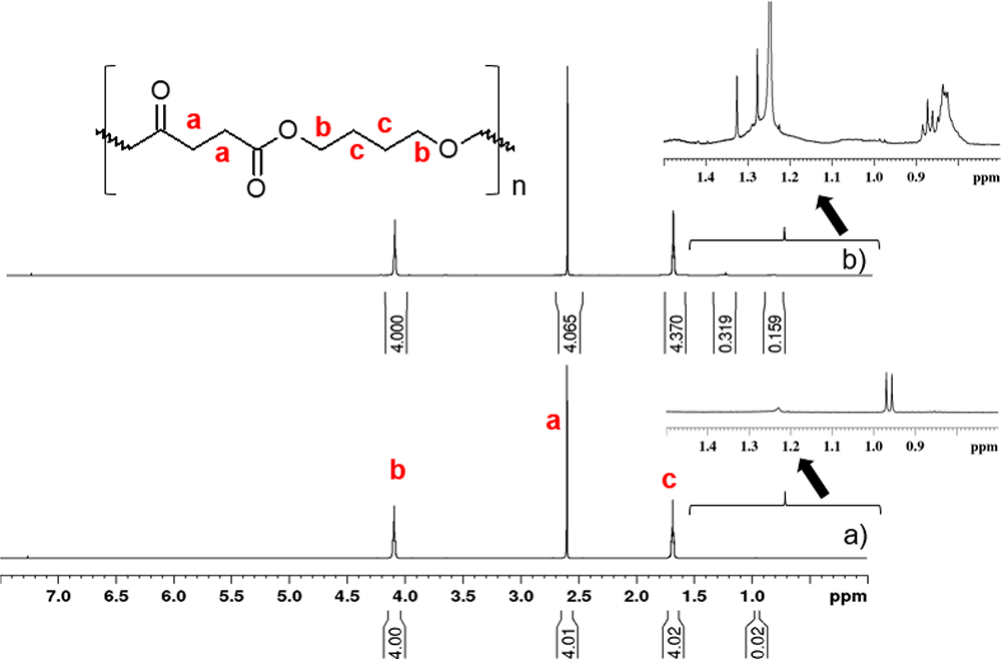
^1^H spectra of pristine (a) and infiltrated (b) PBS. NMR spectra were acquired in chloroform (CDCl_3_) at 298 K. The reported integral values confirmed the accordance of the main signals (a, b, and c) with PBS structure.

The microstructure of pristine and infiltrated PBS samples was investigated by 1D and 2D NMR techniques. ^1^H spectra of pristine and infiltrated PBS are reported in Figure 7. The ^1^H spectrum of pristine PBS sample (Figure 7a) evidenced three main resonances according to the expected polymer structure, which are the methylene group of succinic acid derivative at 2.60 ppm, the methylene group adjacent to oxygen at 4.09 ppm, and the internal methylene group at 1.68 ppm. The PBS structure was confirmed through ^1^H–^13^C Heteronuclear Single Quantum Coherence (HSQC) and ^1^H–^13^C Heteronuclear Multiple Bond Correlation (HMBC) experiments reported in Figure S7 and Figure S8, respectively. After 25 SIS cycles, the ^1^H spectrum of the infiltrated PBS evidenced new resonances as highlighted in the inset of the aliphatic region (0.7–1.4 ppm) in Figure 7b.

To deeply investigate the polymer microstructure and understand the chemical nature of these new resonances deriving from the TMA infiltration on PBS, a series of ^1^H–^13^C multidimensional experiments were conducted. Novel aliphatic groups were identified thanks to analysis of the long-term ^1^H–^13^C correlations in the HMBC spectrum wherein the presence of a cross peak is related to the proton–carbon correlation three or two bonds away. In particular, the HMBC spectrum in Figure S9, shows two distinct groups of signals in the methyl region at 14.13 and 19.76–20.46 ppm, and at 1.1–0.7 ppm in carbon and proton frequencies, respectively, not present in the HMBC spectrum of pristine PBS (Figures S8 and S12), as well as many other resonances assigned to methylene groups in the 25–75 ppm carbon spectral region. Analyzing the long-term correlations in the expanded region of HMBC spectrum of Figure S10, they were assigned to *n*-alkyl groups (i.e., CH_3_(CH_2_)_2_CH_2_–R) and to alkoxyl groups (i.e., CH_3_(CH_2_)_2_CH_2_–O–R). The latter associated with the resonance occurring at 51.9 ppm, a typical chemical shift value for a carbon atom adjacent to the oxygen atom.

**Scheme 1:**
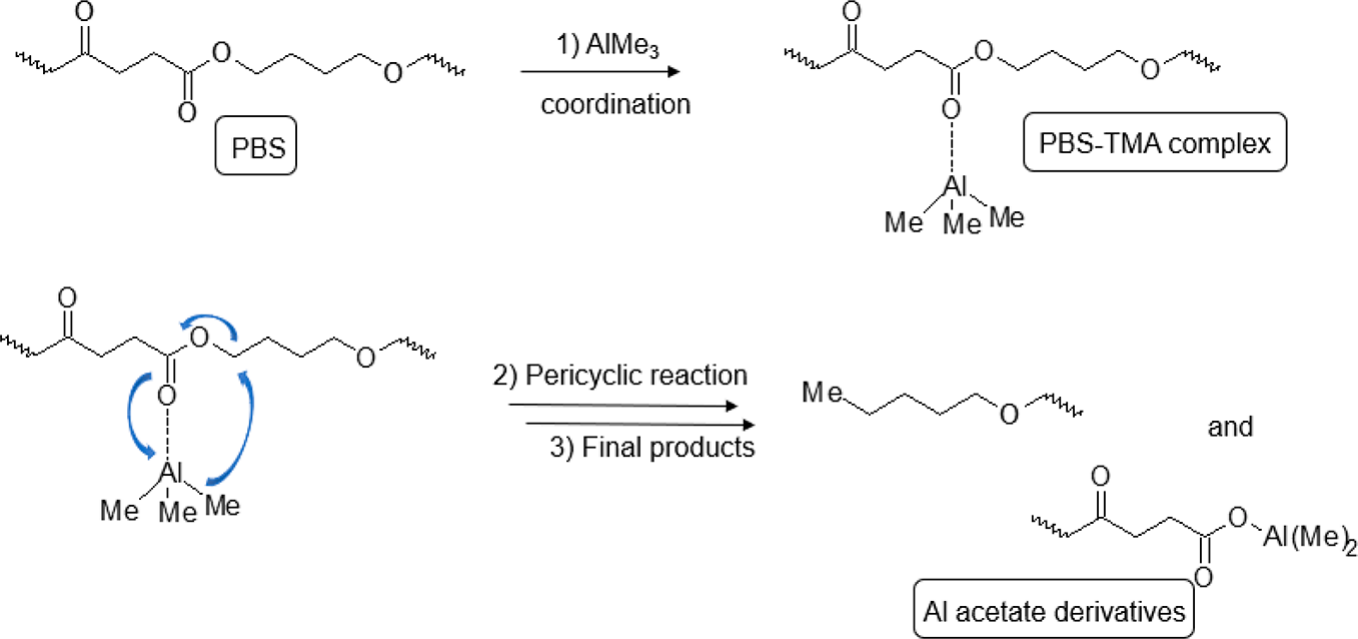
Proposed PBS-TMA Metastable Coordination Complex on Carboxylic Group Leading to Metal Acetate Derivatives

According to these data, a reaction scheme able to justify the new formed aliphatic groups on PBS after TMA infiltration was hypothesized and illustrated in Scheme 1. In the first step, (1), Al of TMA, a Lewis acid, can coordinate the oxygen of the carboxylic group of PBS generating an intermediate coordination complex, (PBS-TMA complex); successively in step (2), a pericyclic reaction takes place thus forming in the step (3) an Al–O bond like a PBS–aluminum acetate complex and a nucleophilic *n*-pentoxy group (CH_3_CH_2_CH_2_CH_2_CH_2_O−) as a leaving group.^43^ It is important to note that TMA is a strong Lewis acid able to perform a nucleophilic insertion into the carbonyl group of PBS, thus giving rise to an aluminum–oxygen–acetate unit as well as other aluminum–oxygen–alkyl units considering that the nucleophilic insertion can occur even on the less favorite position, like oxygen of the ether moiety. Indeed, after hydrolysis, alcoholic (R–OH) and carboxylic acid (R-COOH) moieties were detected, according to the presence of diagnostic signals at 62.3 ppm, (δ_H_ = 3.67 ppm), and 172.7 and 174.2 ppm in the HMBC spectrum of Figure S10. Due to their relevant intensities, these species were excluded as terminal fragments of the main polymer unit. Furthermore, an analysis of PBS end groups was done assigning the hydroxyl end group CH_2_–OH of PBS at 3.7 ppm; the heteronuclear long-range correlations of this end group were not associated with the new formed alkyl- and alkoxy-groups, thus confirming the proposed PBS-TMA reaction mechanism of Scheme 1.^44,45^ To evaluate changes on the PBS microstructure as a function of the number of SIS, a quantitative analysis through ^1^H experiments was performed. Data are reported in Figure S11. Specifically, the relative intensity of the newly formed CH_3_ signals (0.79–0.93 ppm) was measured after normalization of the CH_2_ integral of PBS at 1.73 ppm. In the infiltrated PBS films the amount of the new CH_3_ groups increases from 1.8% to 3.1% upon increasing the number of SIS cycles from 15 to 25, respectively. Interestingly, in the freestanding PBS samples, upon ALD growth of Al_2_O_3_ at 70 °C the amount of the new CH_3_ was measured to be 1.4%, in full agreement with the idea of surface and subsurface growth of Al_2_O_3_ based on the SEM analysis reported in Figure 2. These results are fully consistent with the scission of the polymeric chains due to TMA reaction with C=O groups in the PBS backbone, strongly supporting the reaction mechanism proposed in Scheme 1.

### Polymer Physical Stability

**Figure 8:**
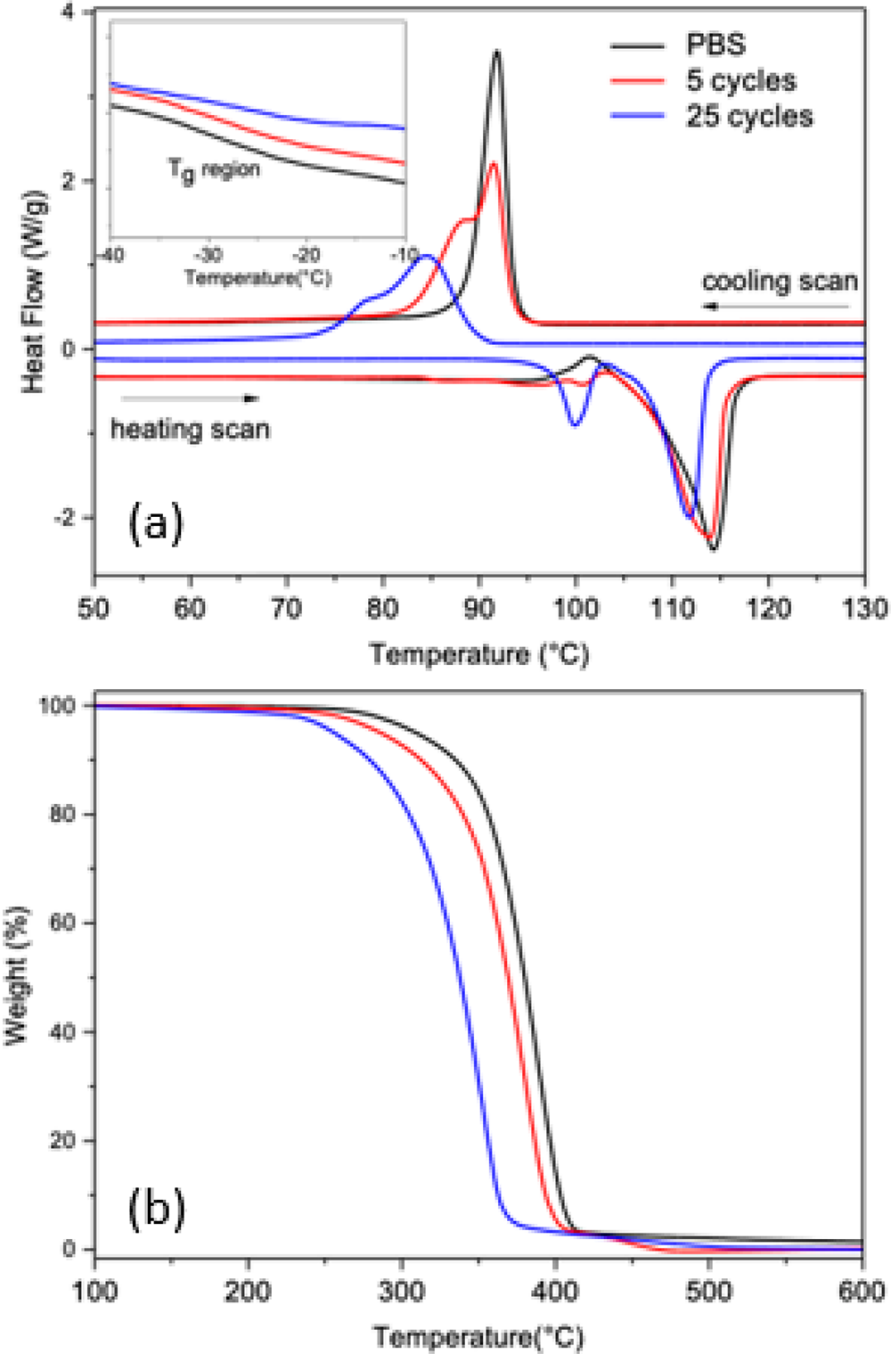
(a) DSC thermograms and (b) TGA curves of pristine (black solid line) and infiltrated PBS films subjected to 5 (red solid line) and 25 (blue solid line) cycles of SIS at 70 °C.

The effect of the Al_2_O_3_ infiltration on the thermal properties of the PBS films was evaluated by DSC and TG. As regards DSC, the first heating scan is more informative concerning the effects of the processing on the properties of materials; therefore the relative thermograms alongside the cooling runs of pristine PBS and infiltrated PBS film samples upon 5 and 25 SIS cycles are reported in Figure 8a. The numerical results of the thermal parameters are listed in Table 1. As shown in the inset of Figure 8a, all curves show a change in heat capacity in the −30 to −25 °C temperature range due to the glass transition (*T*_g_), which increases with the number of SIS cycles. This suggests the occurrence of a gradual stiffening of the polymer amorphous phase due to the restrained mobility of the polymer segments as a consequence of the incorporation of the inorganic phase.^46^ In pristine PBS film, besides the main melting peak (114.2 °C), a cold crystallization peak (101 °C) was noticed, resulting from the melting and subsequent recrystallization of PBS crystals during heating. Notably, increasing SIS cycling caused both temperature and enthalpy of the melting peak to progressively decrease. That is, the massive TMA infiltration partially disrupted the crystalline phase of pristine PBS, causing the formation of a more defective crystalline fraction.^47^ Indeed, after 25 SIS cycles the cold crystallization peak completely disappeared, and a melting signal peaked at 100 °C, with an enthalpy value of about 15 J/g. In the cooling step, pristine PBS exhibited a sharp crystallization peak at about 92 °C, while infiltrated samples showed a multimodal crystallization profile at a comparatively lower temperature (Table 1). This result indicated that the presence of the inorganic phase hindered the PBS crystallization, as well as causing the formation of different crystal lamellae during cooling from the melt. In particular, the crystallization enthalpy of 25 Al_2_O_3_ SIS cycles film was 75% that of pristine polymer, confirming that larger amounts of the inorganic modifier inhibited the PBS crystallization process.

**Table 1:** Thermal Parameters of Pristine an Infiltrated Freestanding PBS Films Obtained from DSC and TG Data

Cycles	*T*_g_ (°C)	*T*_m_ (°C)	Δ*H*_m_ (J/g)	*T*_c_ (°C)	Δ*H*_c_ (J/g)	*T*_5%_ (°C)	*T*_max_ (°C)	Residue_600_ (%)
0	–28.0	114.2	70.0	91.8	72.9	310	389	1.3
5	–26.5	113.9	66.1	91.5	69.9	284	380	0.2
25	–25.8	99.9 (111.8)	15.1 (52.3)	84.5	54.2	250	353	0.05

In Figure 8b, the TGA thermograms of the pristine and infiltrated freestanding PBS films are reported, and the corresponding thermal parameter results are summarized in Table 1. Degradation of PBS occurred in a single weight loss step, and the degradation onset, calculated as the temperature at which 5% weight loss occurred (*T*_5%_), was about 310 °C. Concerning the infiltrated films, a remarkable impairment of thermal stability was observed, as *T*_5%_ dropped to 250 °C after 25 SIS cycles. Correspondingly, the infiltrated systems displayed a significant decrease also in the maximum degradation temperature (*T*_max_) from a value *T*_max_ = 389 °C in the pristine PBS sample to *T*_max_ = 353 °C upon 25 SIS cycles. These outcomes clearly indicate that the SIS process caused cleavage of the polymer chains, decreasing the thermal stability of PBS. Remarkably, no significant differences were noted in the residue at 600 °C, which was almost negligible for all samples. This result suggests the formation of highly volatile polymer–metal compounds, including aluminum alkoxides or carboxylates, which are not fully converted to Al_2_O_3_ and are released in the gas phase before the occurrence of charring.^48^

### Optical Properties

**Figure 9:**
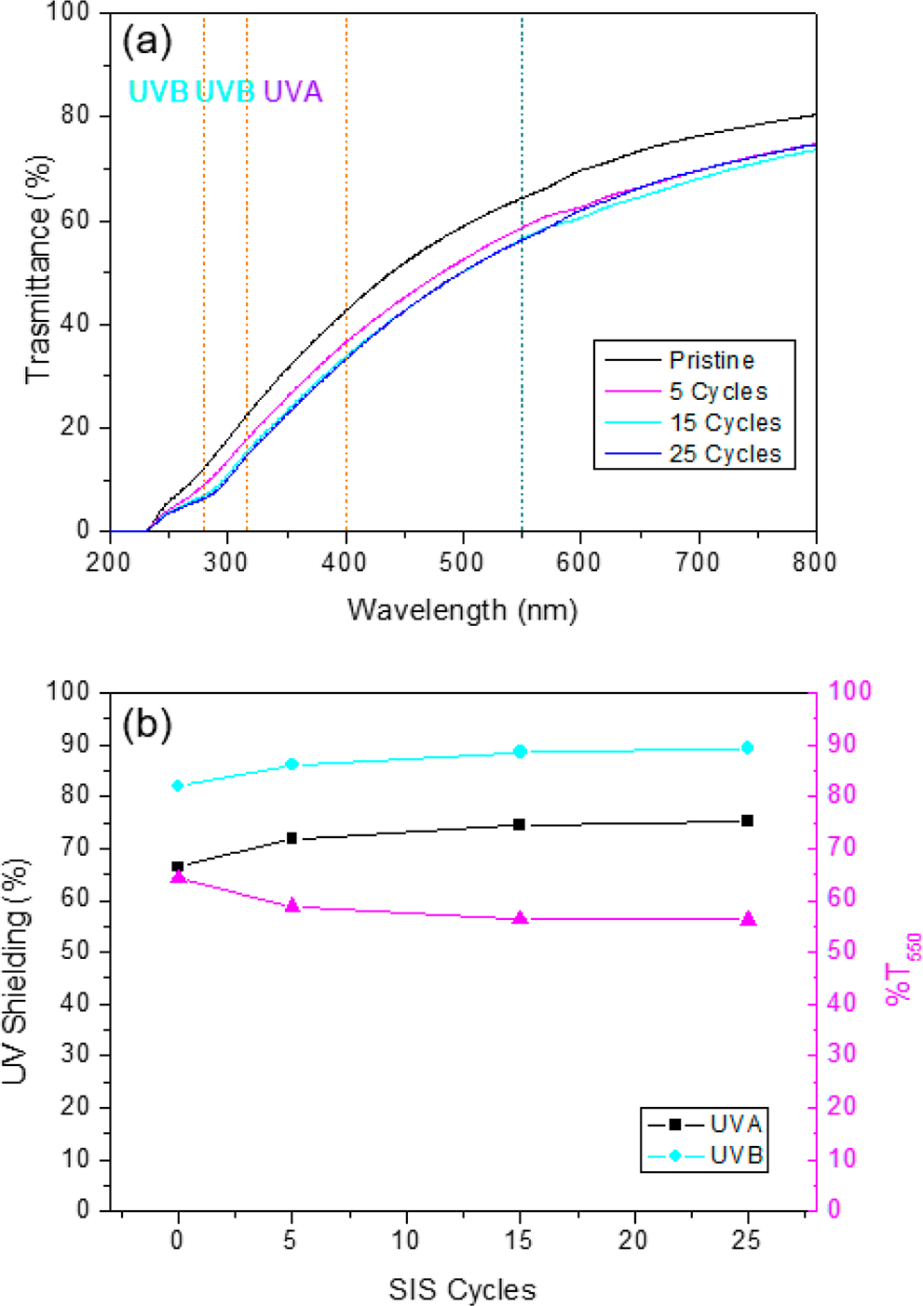
(a) UV–vis light transmission spectra of pristine PBS and infiltrated PBS films upon different numbers of SIS cycles. (b) UVA and UVB-shielding performance and the relative %transmittance at 550 nm of pristine PBS and infiltrated PBS as a function of the SIS cycles. All the samples were infiltrated using a TMA pulse of 0.040 s.

The optical properties of infiltrated PBS films were investigated by UV–vis spectrophotometry and compared with those of pristine PBS matrix. As shown in Figure 9, the pristine PBS matrix is highly transparent and shows poor UVB (280–315 nm) and UVA (315–400 nm) shielding. Figure 9 shows the UV–vis spectra of the PBS films upon 5, 15, and 25 SIS cycles. All the samples were infiltrated using a TMA pulse time *t*_TMA_ = 0.040 s. UV–vis spectrum of spectra of the PBS film upon 5 SIS cycles using a *t*_TMA_ = 0.025 s is reported in Figure S13. Upon infiltration the samples exhibit slightly lower visible light transmission (400–760 nm) and improved UV shielding properties. In particular, increasing the number of SIS cycles and, consequently, increasing the alumina content in the PBS matrix, the samples show a progressive reduction in the visible-light transmission, implying a decrease in transparency of the infiltrated PBS films. As depicted in Figure 9b the transmittance at 550 nm, which gives an estimation of the transparency of the film,^49^ decreases from the initial value of 65% for the pristine PBS sample to 56% for the infiltrated PBS sample upon 25 SIS cycles. Moreover, Figure 9b reports the evolution UVA and UVB shielding performance of the infiltrated PBS as a function of the number of SIS cycles. UVA and UVB shielding were calculated starting from UV–vis spectra in Figure 9a. The protocol for the calculation of the UVA and UVB shielding performances is described in more detail in the Experimental Section. UVA and UVB shielding characteristics increase with the number of Al_2_O_3_ SIS cycles, reaching a sort of saturation above 15 cycles. In particular, the infiltrated PBS samples are found to shield up to 89.4% of UVB radiation and up to 75.2% of UVA radiation, respectively. This variation in UV shielding performances is associated with a slight change of the PBS sample color. Upon SIS, the samples are still transparent, but they are characterized by a pale yellow shade.

## Discussion

Collected data provide important information about the incorporation of Al_2_O_3_ into the PBS matrix by SIS. According to Raman, FTIR and NMR analysis, C=O and −C–O–C– groups are identified as the reactive sites involved in the interaction with the TMA molecules following the scheme proposed in the previous section. It is worth noting that these reactive groups are the same groups that are considered responsible for the trapping of the TMA molecules in the PMMA matrix. Interestingly, the initial density of reactive groups per volume unit in PMMA and PBS is roughly the same, i.e., 1.95 × 10^–5^ and 2.43 × 10^–5^ nm^–3^, respectively. Nevertheless, PBS and PMMA incorporate very different amounts of Al_2_O_3_ when performing SIS with the same experimental conditions. Actually, C=O and −C–O–C– groups are positioned in the backbone chain for PBS and in the side chain for PMMA, as clearly highlighted in Figure 1. These results are perfectly consistent with those reported by Biswas et al.,^41^ who studied the chemical interactions during Al_2_O_3_ SIS process into PMMA and polycaprolactone (PCL), a polyester whose reactive groups are positioned in the backbone chain as in the case of PBS. Interestingly, they reported that nearly 100% of the reactive groups in the PCL matrix react with the TMA molecules, leading to a significant amount of Al_2_O_3_ formed in the first SIS cycle. The C=O group in the backbone structure of PBS and PCL may be more nucleophilic compared to the side chain C=O group in PMMA, resulting in a higher reactivity with Lewis acids with organometallic precursors.^41^ However, there is no obvious evidence in this specific case. Additionally, side chain groups are typically more accessible respect to the backbone groups, suggesting PMMA to be more reactive than PBS when exposed to TMA vapors. All these elements support the idea that the different amount of Al_2_O_3_ that is incorporated in PBS and PMMA during the SIS process at 70 °C is not related to the reactivity of the chemical groups involved in the infiltration process, since they are found to be the same in the two polymers and their density is quite similar in the two polymeric matrices.

Accordingly, as proposed by Leng and Losego,^16^ we can speculate that the differences between PMMA and PBS are directly correlated to the physiochemical features of precursors/polymers systems and in particular to the different free volume of the two polymers and to the burial of the free volume. First of all, we have to take into account the very different glass transition temperatures of the two polymers, with *T*_g_ ∼ 115 °C for PMMA and *T*_g_ ∼ −28 °C for PBS, respectively. Infiltration process was performed at 70 °C, i.e., well above *T*_g_ for PBS and below *T*_g_ for PMMA. In this latter condition, the backbone of the PMMA polymer is rigid and behaves like a glassy polymer with a reduced free volume. Conversely, in PBS the polymer chain segmental motions of the flexible aliphatic components in the continuous polymer backbone determines a change in the free volume of polyester. As a consequence, at this temperature, PBS behaves like a rubbery polymer with a large free volume. Accordingly, the high amount of Al_2_O_3_ incorporated into the PBS matrix could be due to enhanced sorption and diffusion of TMA molecules associated with the large free volume of PBS at 70 °C. Similar considerations hold in the case of PCL that has a glass transition temperature *T*_g_ ∼ −60 °C. Padbury et al.^50^ demonstrated that the *T*_g_ of a polymer affects the absorption of TMA molecules. In particular, they proposed a simple jump diffusion model: according to this model, diffusion of solute molecules depends on the probability of finding a critical free volume void to accommodate the solute molecules. Accordingly, the temperature is reported to be a critical parameter for the diffusion of solute molecules in the polymer matrix. In particular, below *T*_g_, the diffusion coefficient is significantly reduced.^51^ In this respect, the reaction mechanism proposed by NMR analysis is perfectly consistent with this hypothesis, because the cleavage of the chains that are supposed to occur due to the reaction with the TMA molecules leads to an increase of free volume, further increasing the possibility to incorporate Al_2_O_3_ into the PBS matrix. Experimentally, Padbury and Jur^52^ demonstrated that inorganic mass uptake of organometallic precursors from poly *n*-methacrylate polymers depends on the size of polymer side groups. In particular, TMA diffusion was inferred to decrease monotonically with increasing side group length due to the creation of a tortuous network of interconnected pores that hinders the TMA diffusion. Accordingly, we speculate that tortuosity of the polymer network represents a limiting factor to sorption and diffusion of TMA in PMMA with respect to PBS.

In addition, as already highlighted, the proposed reaction scheme during the SIS process in PBS implies the scission of the polymeric chains. This scheme is perfectly consistent with results reported by Gong and Parsons,^53^ hypothesizing the chain scission of a polyester with a reactive group on the main polymeric chain during the SIS process. Accordingly, the infiltrated PBS matrix exhibit reduced thermal and mechanical stability, making this hybrid material extremely brittle when increasing the Al_2_O_3_ mass uptake. This significantly impacts the effective possibility to follow this approach in order to tune the optical properties of PBS and improve the shelf life of goods when using modified PBS for packaging. To avoid the polymeric chain scission the reactive group should be incorporated in the polymer molecule as side groups, like in PMMA. In this respect, the introduction of a small number of reactive sites incorporating MMA monomers into a nonreactive polymer like polystyrene (PS) is enough to guarantee Al_2_O_3_ incorporation, as demonstrated in a previous paper by Caligiore et al.^54^ The present results identify important constraints that limit the effective possibility to incorporate inorganic materials into some polymers by SIS, suggesting at the same time a viable strategy to overcome these limitations.

Additionally, present data demonstrate that infiltration of Al_2_O_3_ into bio-based and biodegradable PBS films is possible, but, irrespective of the high amount of Al_2_O_3_ that is incorporated in the PBS, a quite limited improvement of the UV shielding properties is registered, in conjunction with a decrease of the film transparency. In addition, the film becomes extremely brittle limiting the possibility of using the Al_2_O_3_ infiltrated PBS film for packaging applications. In this respect, the data provided clearly demonstrate that this approach does not provide a viable solution to tailor optical and mechanical properties of this specific polymer. From a more general point of view, our experimental findings clearly point out an important limitation that has to be taken into account for synthesis of hybrid organic–inorganic materials through the incorporation of an inorganic material into a polymeric matrix by the SIS process. For polymer having the reactive group on the main polymeric chain, incorporation of the inorganic phase implies a progressive degradation of the mechanical properties of the final hybrid organic–inorganic material. From another point of view, present results suggest that PBS could represent an effective polymer matrix for the incorporation of alumina and the formation of Al_2_O_3_ nanostructures when used as a block in a self-assembled di- or ter-block copolymer template like in the case of a more conventional PS-*b*-PMMA system.^55^ Incorporation of alternative metal oxides could be explored as an alternative solution to further improve the UV shielding properties of the PBS films. Proper selection of the titanium precursor could guarantee the possibility to infiltrate into the PBS matrix operating at low temperature; nevertheless the effective capability to improve the optical properties of this polymer is questionable, because the amount of TiO_2_ incorporated into the PBS has to be limited in order to avoid significant degradation of its thermal and mechanical properties.

## Conclusion

In summary, Al_2_O_3_ growth in PBS freestanding films (∼30 μm) using a sequential infiltration synthesis process via TMA and H_2_O as precursors at 70 °C was investigated. Through rational tuning of the SIS processing parameters, homogeneous growth of Al_2_O_3_ throughout the entire PBS film thickness is possible. Detailed characterization of infiltrated PBS revealed chemical and physical modification of the films in terms of reduced thermal stability and increased UV shielding. NMR and FTIR spectroscopies revealed the interaction of the TMA precursor with the ester groups in the polymeric chains inducing a chain scission that significantly affects the mechanical properties of the PBS film. Infiltration of Al_2_O_3_ into PMMA and PBS thin films showed that Al_2_O_3_ incorporation in PBS is much higher than in PMMA due to the different free volume of the two polymers at 70 °C. To conclude, this work provides important information about the reaction mechanism of TMA during the infiltration process, which will offer the opportunity to expand the library of polymers that can be infiltrated by SIS at low process temperature.

## Supporting Information Available

Thermal treated PBS Raman spectra, XPS survey spectra of pristine and treated freestanding PBS, Initial thickness and refractive index of pristine PBS spin-cast samples, Raman peaks intensities, Change in FTIR peaks, ^1^H–^13^C HSQC spectrum of pristine PBS film, ^1^H–^13^C HMBC spectrum of pristine PBS film, ^1^H–^13^C HMBC spectrum of processed PBS film, ^1^H–^13^C HMBC spectrum of processed PBS film, Comparison of HNMR spectra, ^1^H–^13^C HMBC spectrum comparison of pristine and treated PBS film, UV–vis transmittance spectra of SIS treated PBS films (PDF)
