# Supplementary material for: Sequential Infiltration Synthesis of Al2O3 in Biodegradable Polybutylene Succinate: Characterization of the Infiltration Mechanism
Source: ACS Appl Polym Mater. 2022 Oct 3;4(10):7191–203. doi: 10.1021/acsapm.2c01073 (PMC9578113; doi:10.1021/acsapm.2c01073)
Supplement: Supplementary file 1 — ap2c01073_si_001.pdf [file ap2c01073_si_001.pdf]

Supporting Information:

Sequential Infiltration Synthesis of Al_2O_3 in Bio-Degradable Polybutylene Succinate: Characterization of the Infiltration Mechanism

Alessia Motta[1,2], Gabriele Seguini[1], Michele Perego[1,], Roberto Consonni[3], Antonella Caterina Boccia[3,*], Gina Ambrosio[4], Camilla Baratto[4], Pierfrancesco Cerruti[5], Marino Lavorgna[5], Stefano Tagliabue[6] and Claudia Wiemer[1]*

[1] CNR-IMM, Unit of Agrate Brianza, Via C. Olivetti 2, I-20864 Agrate Brianza, Italy

[2] Department of energy, Politecnico di Milano, Via Ponzio 34/3, 20133 Milano, Italy

[3] CNR- SCITEC, Via A. Corti 12, I-20133 Milano, Italy

[4] CNR-INO, PRISM Lab, Via Branze 45, 25123 Brescia, Italy

[5] CNR-IPCB, Via G. Previati 1/E, 23900 Lecco, Italy

[6] Corapack srl, Via del Fontaline 7, 22040 Brenna, Italy

*Email: michele.perego@cnr.it

*Email: antonella.boccia@scitec.cnr.it

EXPERIMENTAL METHODS

Raman spectroscopy. Raman measurements were recorded with an excitation wavelength of 785 nm using a confocal micro-Raman setup (Horiba) equipped with and a Peltier cooled CCD detector. An Olympus microscope with an objective of 50X was used during the experiments. The signal was dispersed with 600 grooves/mm grating. The laser power impinging on the sample was about 5 mW. The spectral calibration was performed using a Si substrate. In order to avoid Raman signal from microscope glass support, a silicon substrate was used below all the PBS samples. The removal of the baseline in the Raman spectra was done through the LabSpec 6 software of the Horiba system. Since the polymers are transparent to 785 nm, the signal observed in the Raman spectrum was collected from the whole thickness of the biopolymer and not only from the region focused on the sample.

X-ray photoemission spectroscopy. The films were inspected by X-ray photoemission spectroscopy (XPS) on a PHI 5600 system equipped with a monochromatic Al Kα source (1486.6 eV) and a concentric hemispherical analyzer. Electron gun was used to mitigate charging phenomena during analysis of the infiltrated freestanding PBS film.

Scanning Electron Microscopy. SEM images of infiltrated film cross-sections were acquired using a FEI Quanta 200 FEG electron microscope, with a 10-30 kV acceleration voltage, and secondary electron detector (FEI, Eindhoven, The Netherlands). Energy dispersive X-ray (EDX) mapping analysis was performed on the film cross sections by means of the above-mentioned SEM equipped with an Inca Energy System 250 and an Inca-X-act LN2-free analytical silicon drift detector (Oxford Instruments, Abingdon-on-Thames, UK)

Spectroscopic Ellipsometer. The ellipsometric data were collected by using a rotating compensator ellipsometer equipped with Xe lamp (M-2000F, J. A. Woollam Co. Inc.) in a wavelength range of 250-1000 nm at a fixed 75° incidence angle. Ellipsometric data were analyzed by EASE software package

2.3 version (J. A. Woollam Co. Inc.). To estimate polymer layer thickness ellipsometric data were fitted using a film stack model composed of a Cauchy layer model on SiO_2 on the silicon substrate in the wavelength of 400- 1000 nm, because of the absorbing nature of the ester group in the ultraviolet range. The silicon dioxide thickness was previously acquired before spin casting for each sample to better fit the Cauchy model after the spin process.

Fourier transform infrared spectroscopy. FTIR-ATR spectra of the films were acquired by means of a Perkin Elmer Spectrum 100 spectrophotometer (USA), equipped with a Universal ATR diamond crystal sampling accessory. Spectra were recorded as an average of 16 scans, with a resolution of 4 cm^{-1}

Thermal Analysis. The thermal properties of the PBS-based films were assessed by differential scanning calorimetry (DSC), using a DSCQ1000-TA Instrument (New Castle, DE, USA) with a scan rate of 10 °C/min from -50 to 170 °C). Thermal degradation of the samples was studied by thermogravimetry (TG) under air, using Q500-TA Instrument thermobalance (New Castle, DE, USA), with a scan rate of 10°C/min in the 25-800 °C temperature range.

Nuclear magnetic resonance spectroscopy: Samples were dissolved in Chloroform (CDCl3-d) and/or Tetrachloroethane (TCE-d4) deuterated solvents. Spectra were acquired on Bruker 600 Neo NMR spectrometer operating at 14.1 T, equipped with 5 mm cryoprobe PRODIGY and using TOPSPIN 4.1.3 version. Proton and carbon monodimensional experiments were acquired with 9000 and 36000 Hz, respectively, over 64 K data points each. Multidimensional homonuclear ^{1}H-^{1}H Total Correlation Spectroscopy (TOCSY), ^{1}H-^{13}C Heteronuclear Single Quantum Coherence (HSQC), and ^{1}H-^{13}C Heteronuclear Multiple Bond Correlation (HMBC) experiments were performed with the non-uniform sampling mode (NUS) activated. Spectra were calibrated according to solvent chemical shift ($CHCl_3$ at 7.27 ppm, TCE at 6.95.

UV–visible spectrophotometry: The transmission of light through the samples was measured with a Cary 5000 UV-Vis-NIR spectrometer (Agilent Technologies) in a dual-beam configuration, covering a frequency range from 3500 to 50000 cm^{-1}. The experimental data were used to calculate the absorbance of the different samples. The UV absorption properties of PBS/Al_2O_3 films were investigated by absorption spectroscopy using UV−vis spectra [27]. The blocking percentage for UV-A (320–400 nm) was calculated by equation 1:

$$1)\ \ \mathrm{UVA - blocking\ (\%)} = 100 - \frac{\int_{320}^{400} \mathrm{T(\%)d\lambda}}{\int_{320}^{400} \mathrm{d\lambda}}$$

Accordingly, the blocking percentage for UV-B (280–320 nm) was calculated by equation 2:

$$2)\ \ \mathrm{UVB - blocking\ (\%)} = 100 - \frac{\int_{280}^{320} \mathrm{T(\%)d\lambda}}{\int_{280}^{320} \mathrm{d\lambda}}$$

where T(λ) is average spectral transmittance, dλ is bandwidth, and λ is the wavelength.

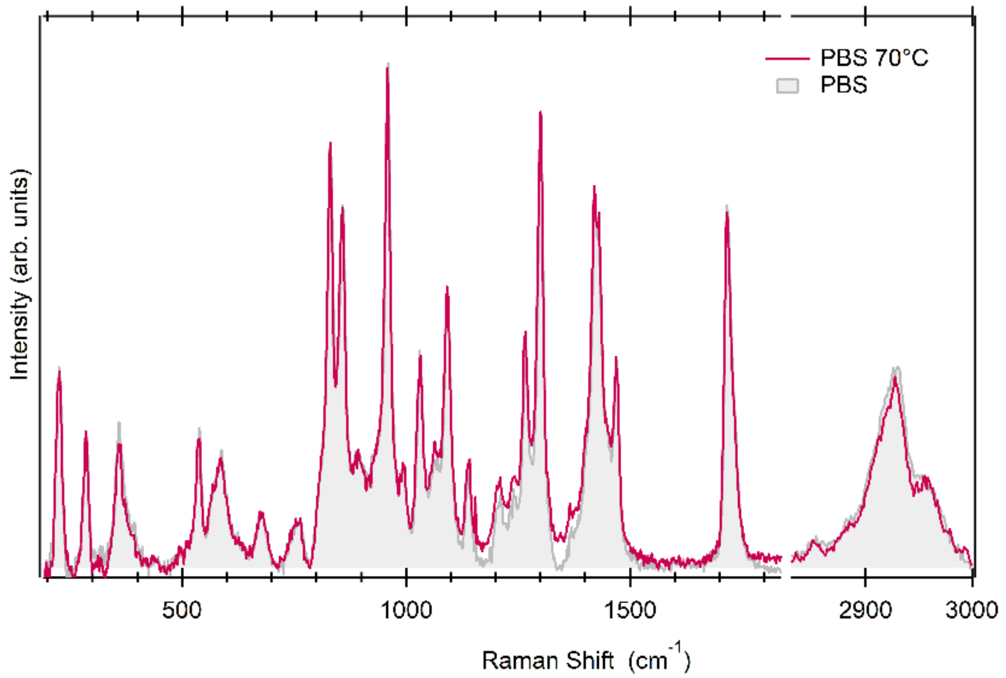

Figure S1. Raman spectra of pristine PBS and part of pristine PBS of the infiltrated sample (t = 0.040s) and heated up to 70°C.

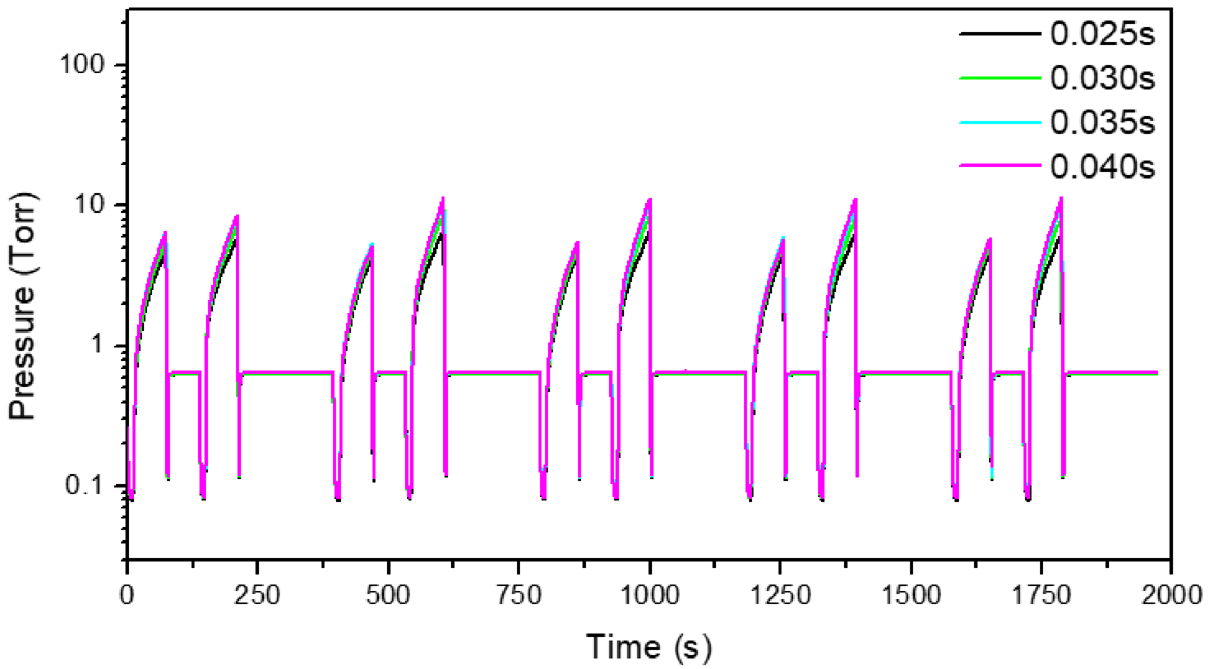

Figure S2. Chamber pressure during Al_2O_3 SIS process at different TMA pulse time.

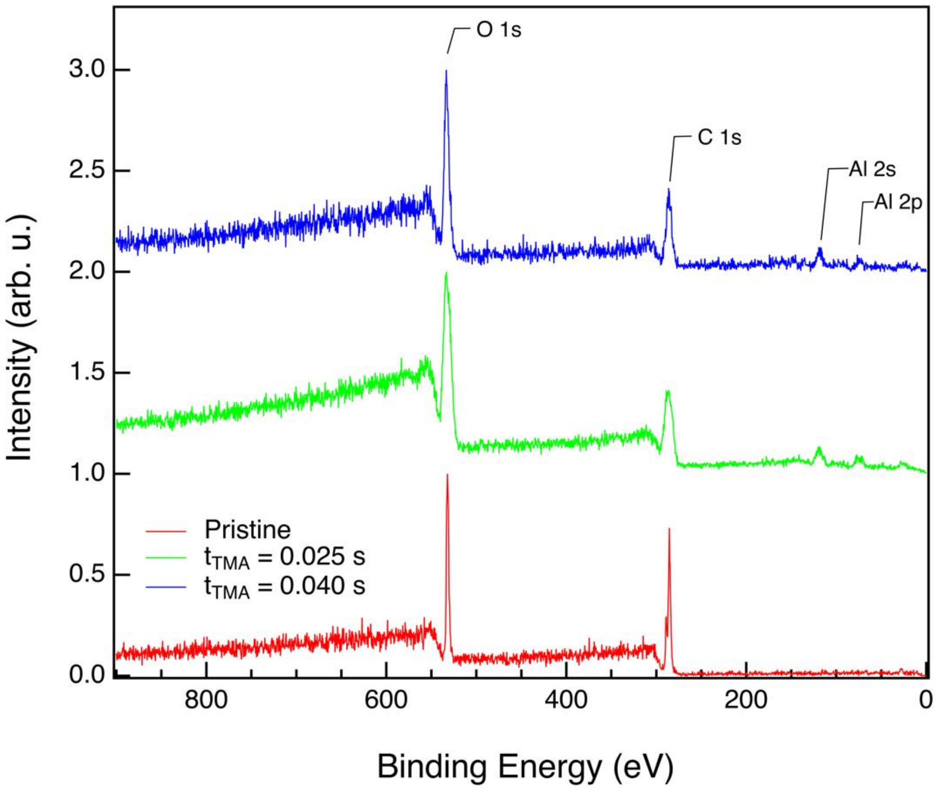

Figure S3. XPS survey spectra of pristine (red line) and infiltrated PBS films. Infiltrated PBS samples were processed with 5 SIS cycles at two different TMA pulse time of 0.025 s (green line) and 0.040 s (blue line).

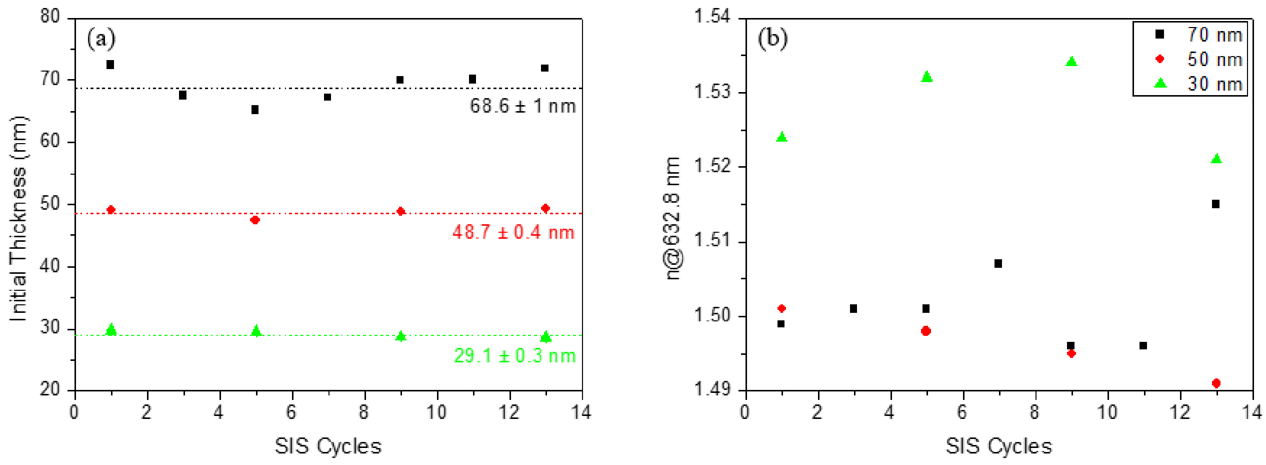

Figure S4. a) Variation on the initial thickness of polymer samples. b) Variation of the pristine PBS refractive index at 632.8 nm.

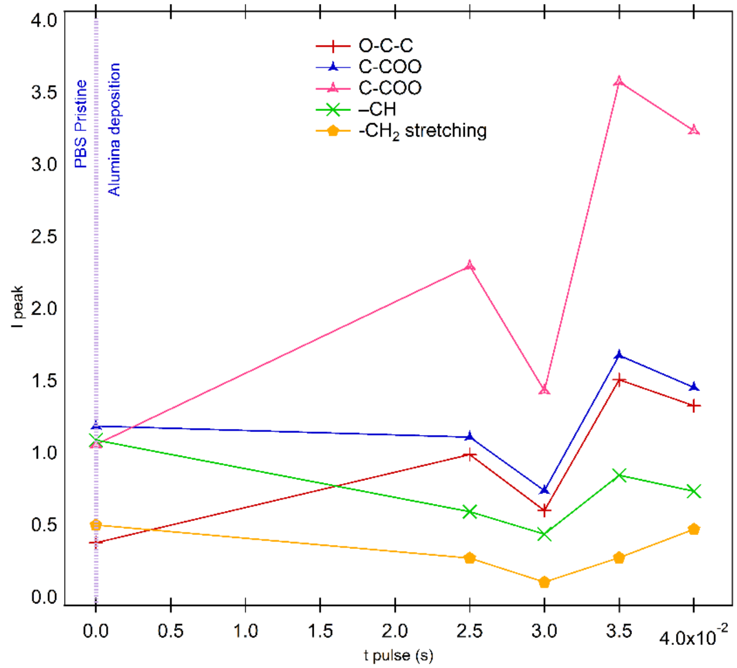

Figure S5. Intensities of the major Raman peaks with respect to TMA pulse times

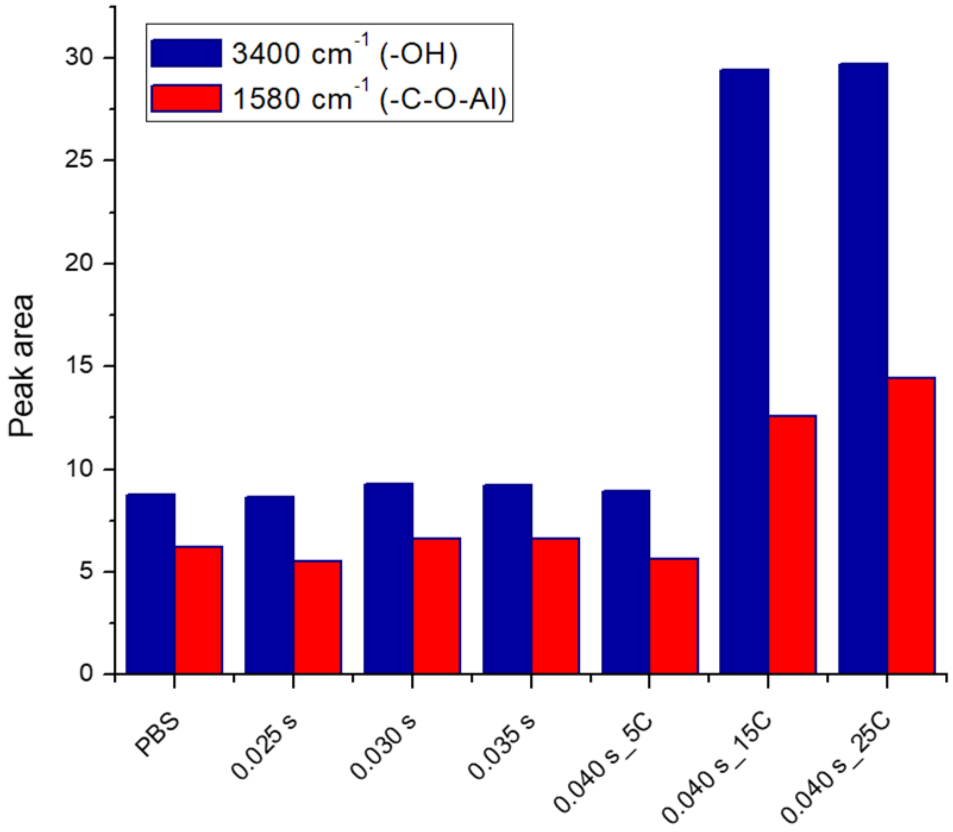

Figure S6. Changes in Al-O-C peak at 1580 cm-1 and the O-H band at 3400 cm^{-1} in SIS infiltrated PBS films at different pulse time and cycle numbers.

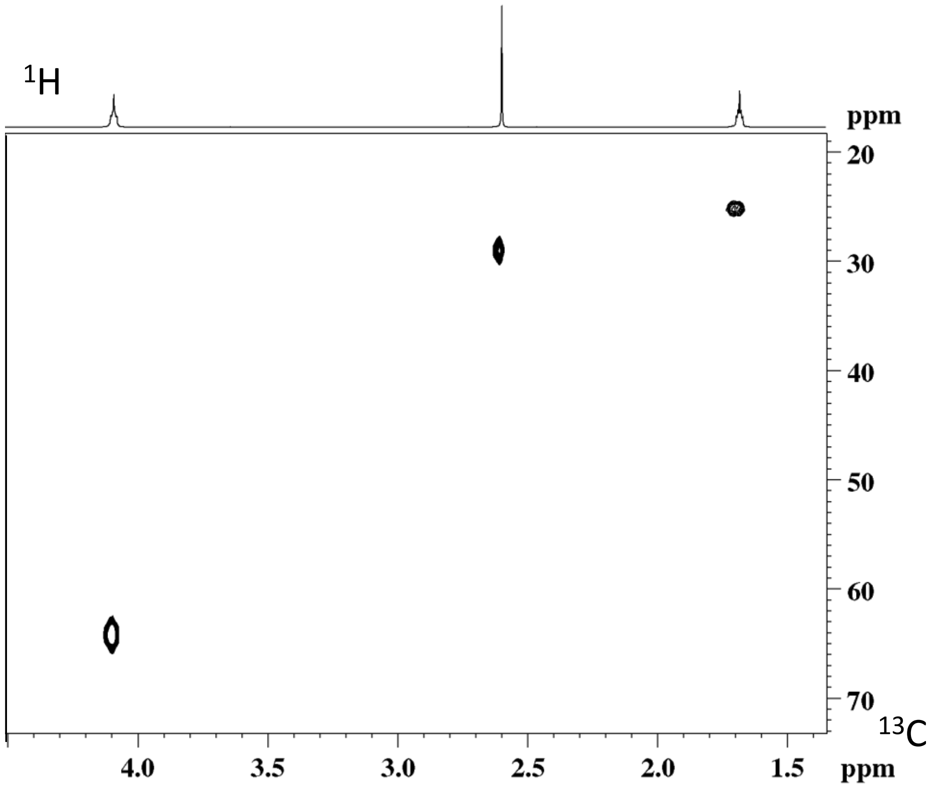

*Figure **S7**: ^{1}H-^{13}C HSQC spectrum of pristine PBS in chloroform at 298 K.*

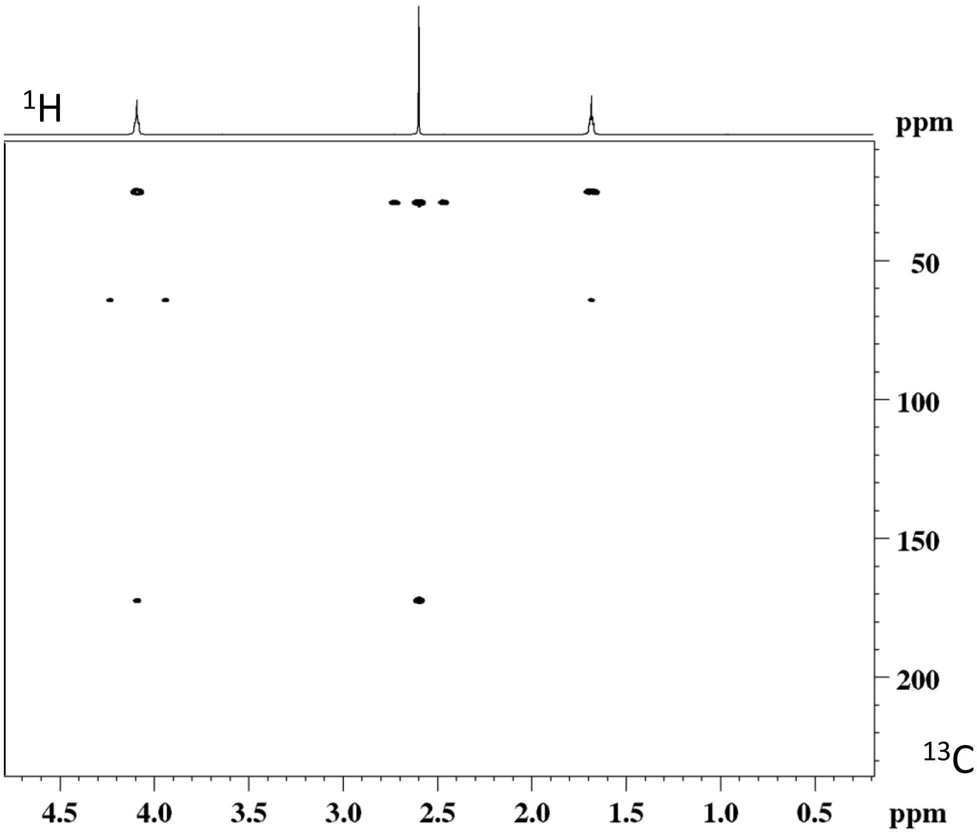

Figure S8: *^{1}H-^{13}C HMBC spectrum of pristine PBS chloroform at 298 K.*

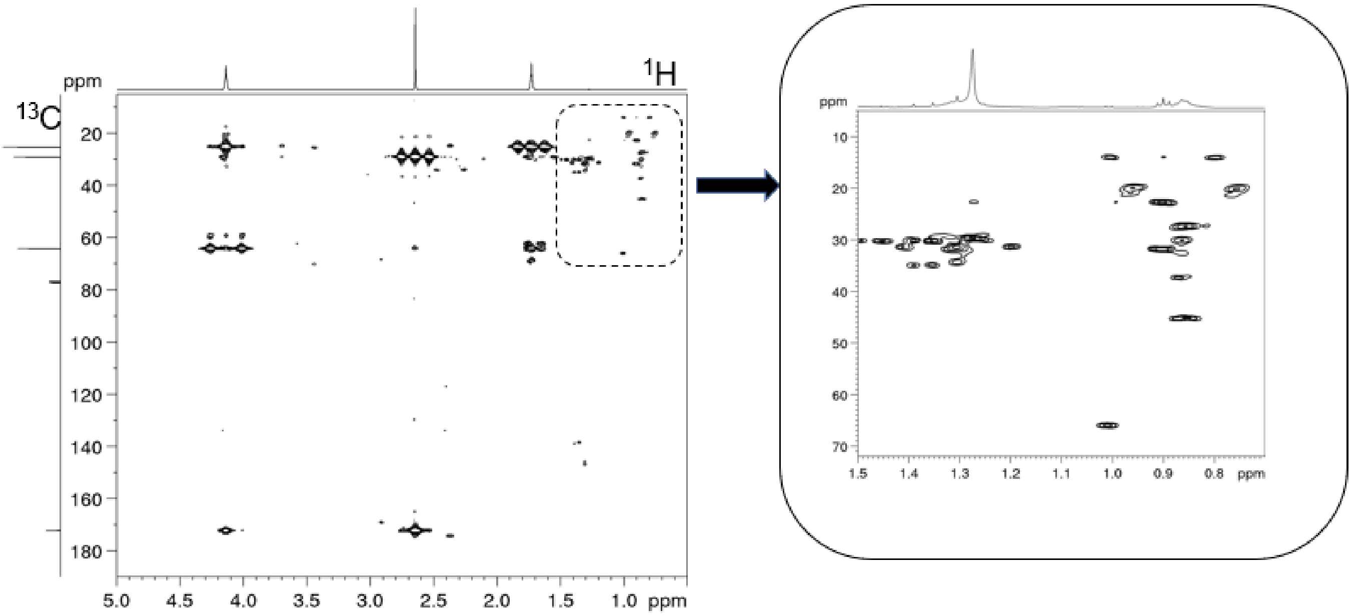

Figure S9. ^{1}H-^{13}C HMBC spectrum of infiltrated PBS upon 25 SIS cycles in chloroform at 298 K. The inset on the right is an expanded region of full HMBC spectrum.

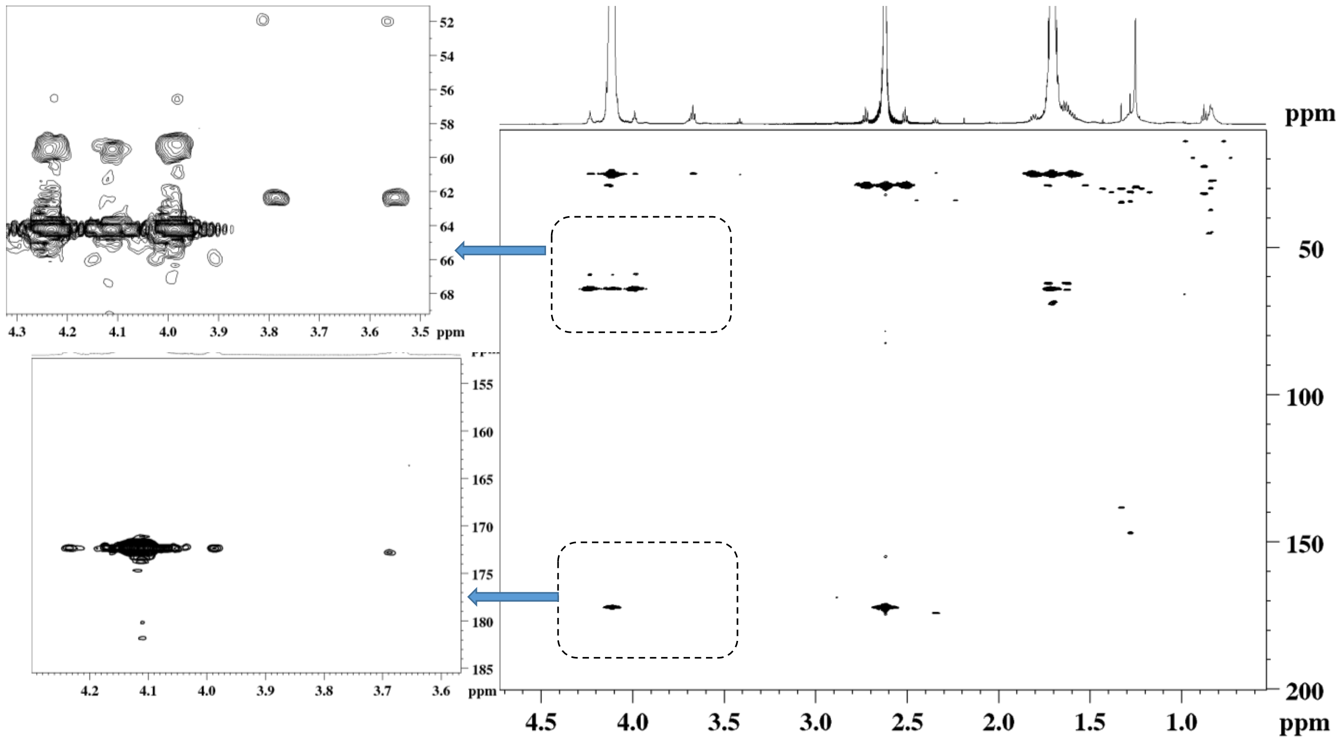

Figure S10: *^{1}H-^{13}C HMBC spectrum of infiltrated PBS film. The sample underwent 25 SIS cycles with a TMA pulse time of 0.040 s. Zooms on two selected spectral regions are reported ion the left. The experiment was acquired in chloroform at 298 K.*

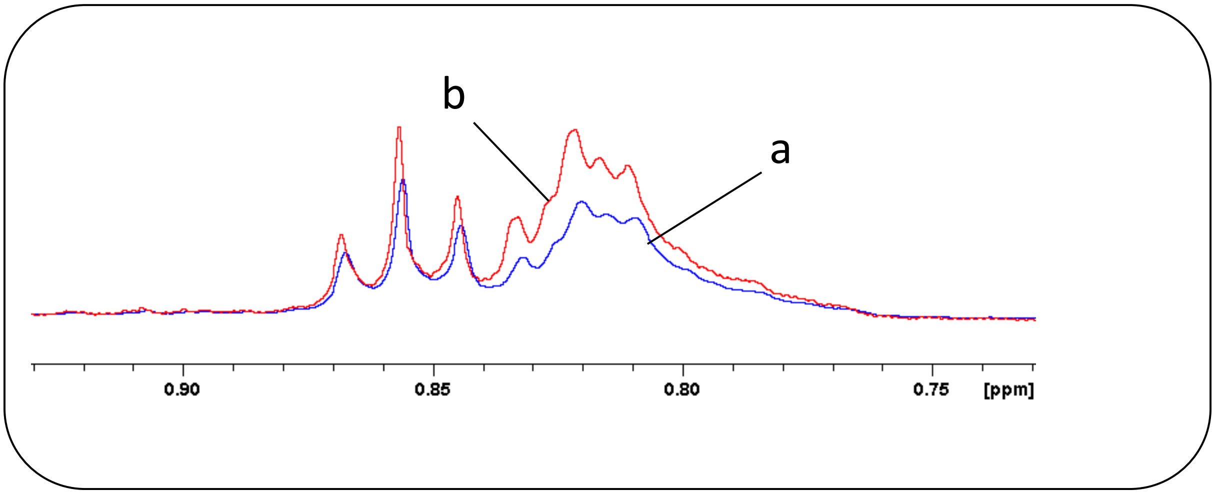

Figure S11: *^{1}H spectra of freestanding PBS samples upon infiltration of Al_2O_3 with 15 a) and 25 b) SIS cycles recorded in chloroform at 298 K. TMA pulse time of 0.040 s was fixed for both samples.*

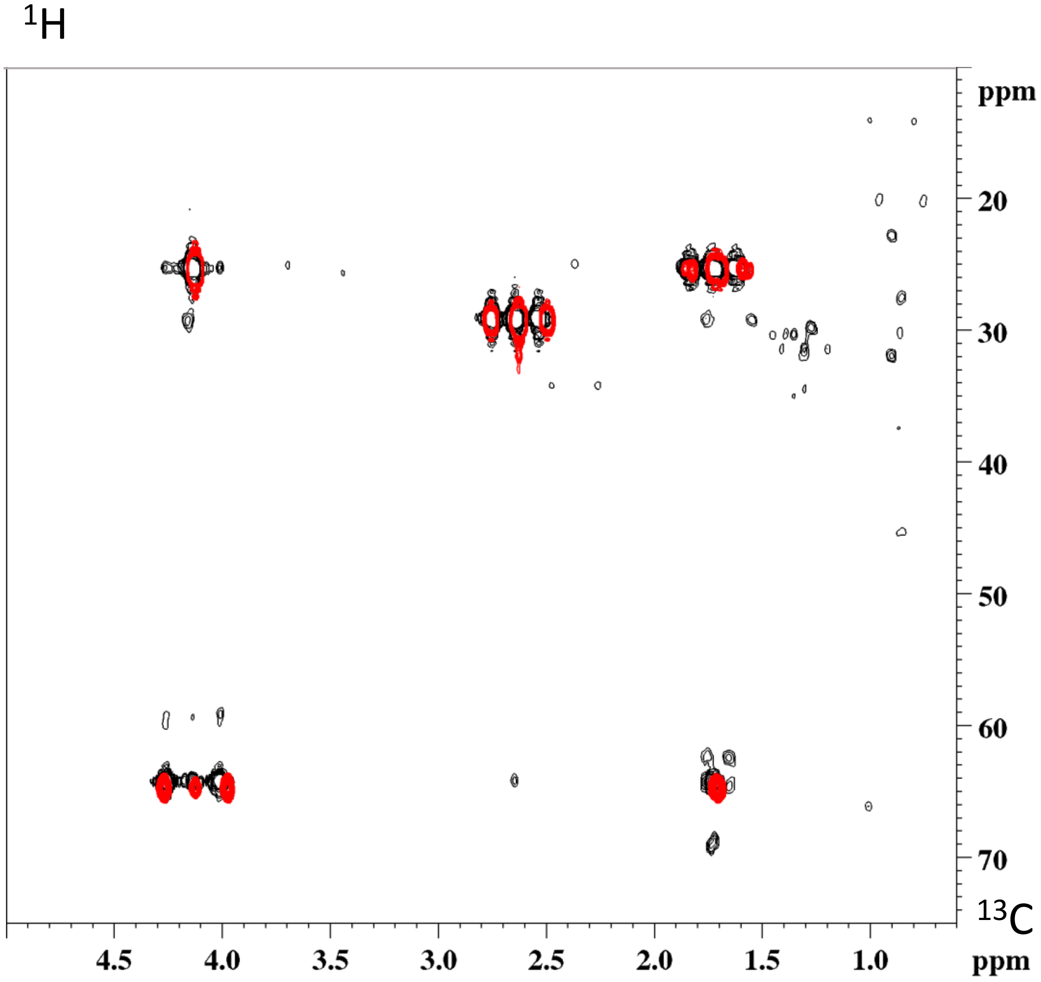

Figure S12. *Comparison of ^{1}H-^{13}C HMBC spectra of pristine PBS sample (in red) and after TMA infiltration (in black). Spectra were recorded in chloroform at 298 K.*

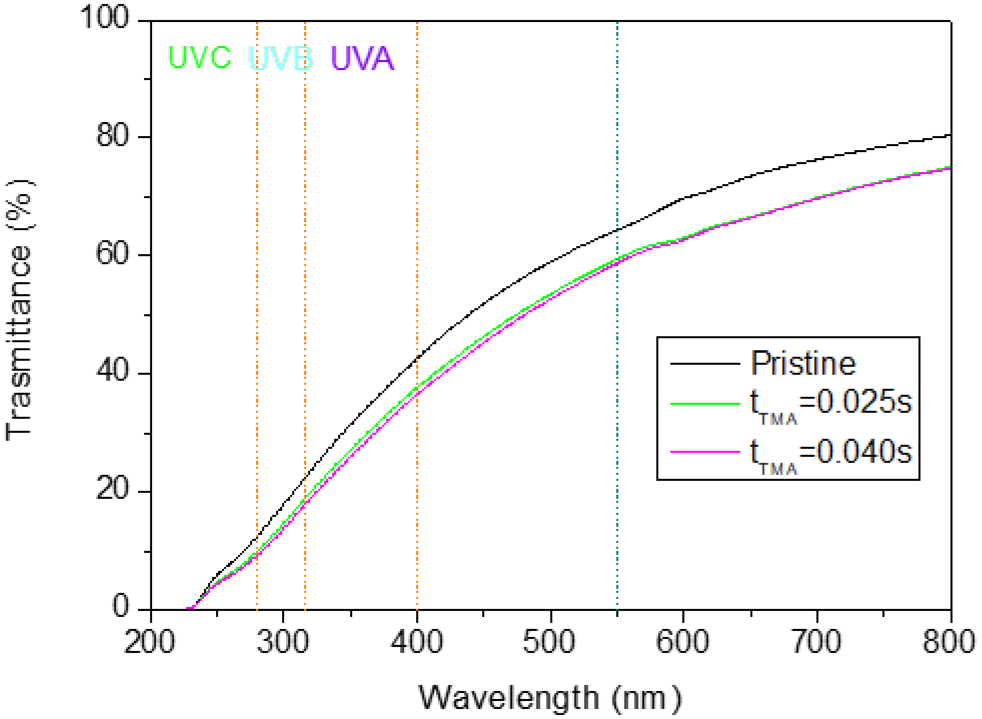

Figure S13. UV–vis light transmittance spectra of pristine PBS and 5 Al_2O_3 SIS cycles PBS films upon different TMA pulse time.
